# Diversification of Phage-Displayed Peptide Libraries with Noncanonical Amino Acid Mutagenesis and Chemical Modification

**DOI:** 10.1021/acs.chemrev.4c00004

**Published:** 2024-04-30

**Authors:** J. Trae Hampton, Wenshe Ray Liu

**Affiliations:** † Texas A&M Drug Discovery Center and Department of Chemistry, College of Arts and Sciences, Texas A&M University, College Station, Texas 77843, United States; ‡ Institute of Biosciences and Technology and Department of Translational Medical Sciences, College of Medicine, Texas A&M University, Houston, Texas 77030, United States; § Department of Biochemistry and Biophysics, College of Agriculture and Life Sciences, Texas A&M University, College Station, Texas 77843, United States; ∥ Department of Cell Biology and Genetics, College of Medicine, Texas A&M University, College Station, Texas 77843, United States; ⊥ Department of Pharmaceutical Sciences, Irma Lerma Rangel College of Pharmacy, Texas A&M University, College Station, Texas 77843, United States

## Abstract

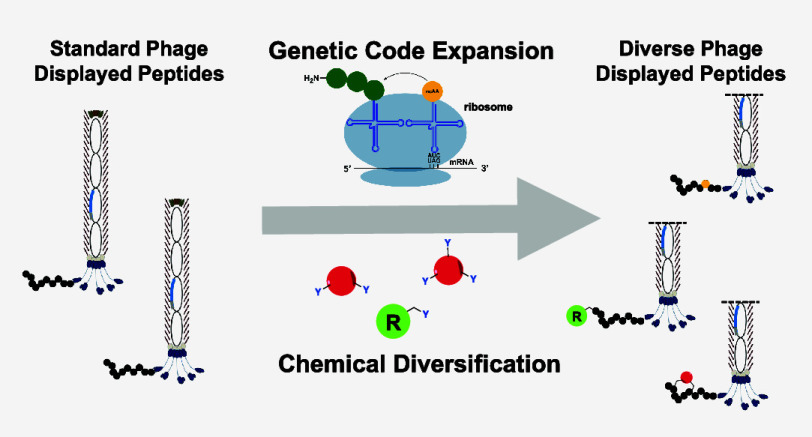

Sitting on the interface between biologics and small molecules, peptides represent an emerging class of therapeutics. Numerous techniques have been developed in the past 30 years to take advantage of biological methods to generate and screen peptide libraries for the identification of therapeutic compounds, with phage display being one of the most accessible techniques. Although traditional phage display can generate billions of peptides simultaneously, it is limited to expression of canonical amino acids. Recently, several groups have successfully undergone efforts to apply genetic code expansion to introduce noncanonical amino acids (ncAAs) with novel reactivities and chemistries into phage-displayed peptide libraries. In addition to biological methods, several different chemical approaches have also been used to install noncanonical motifs into phage libraries. This review focuses on these recent advances that have taken advantage of both biological and chemical means for diversification of phage libraries with ncAAs.

## 1 Introduction

### 1.1 Peptide Therapeutics

**Figure 1:**
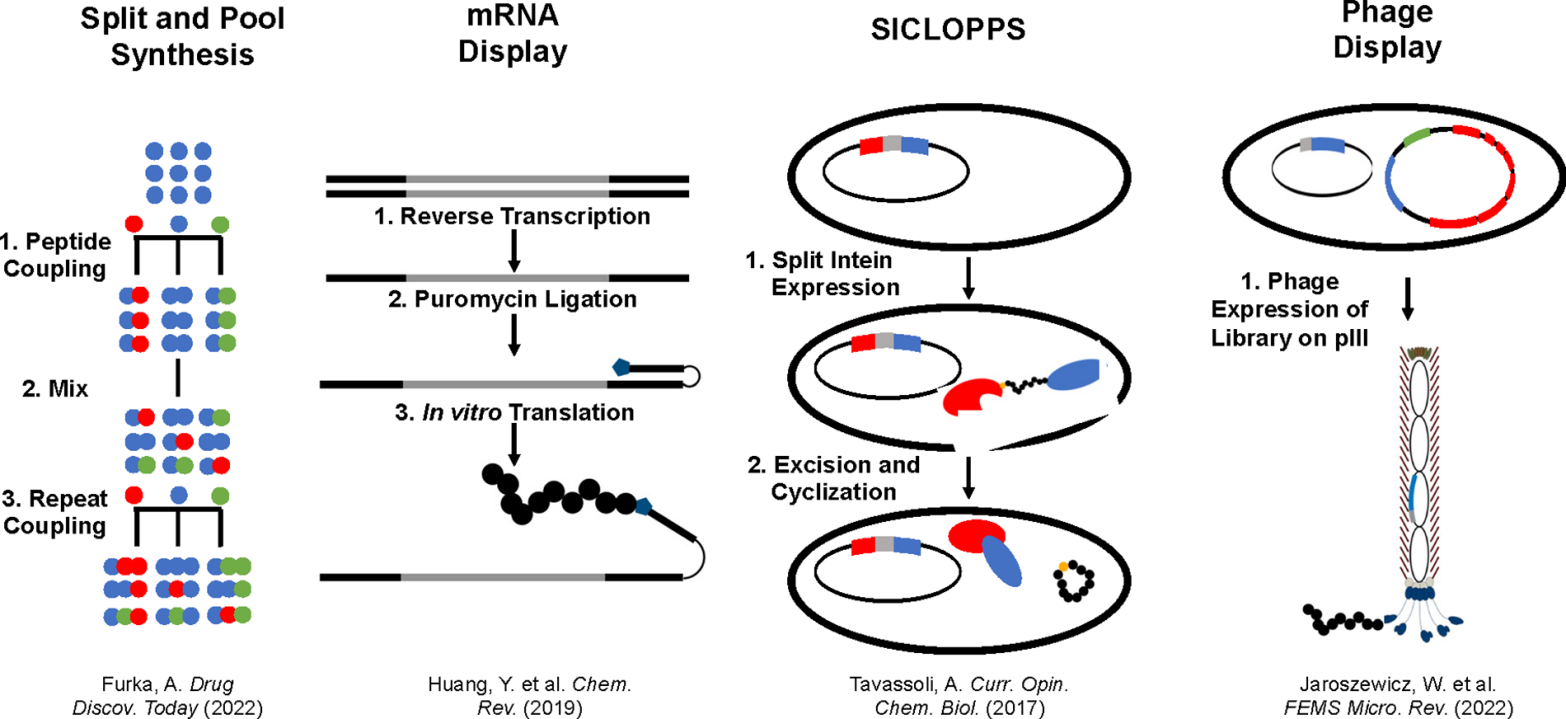
Overview of techniques for peptide library generation. Several methods are currently available for the generation of large peptide libraries. These include completely synthetic methods (Split and Pool), *in vitro* methods (mRNA Display), and strictly *in vivo* methods (SICLOPPS). Phage display gives the possibility to generate libraries *in vivo* but perform selections in a variety of different conditions. Techniques that are not illustrated in the figure but widely used are yeast display, DNA-encoded peptide library technique, one-bead-one-compound peptide library technique, etc.

Peptides, or short protein fragments with a molecular weight less than 5000 Da, occupy an intriguing space in drug discovery, as they have the potential to combine the specificity of large biologics (antibodies, proteins, etc.) with the low cost of production, storage, and administration of small molecules. Due to their larger size, peptides are efficient at inhibiting protein–protein interactions, which small molecules generally are ineffective in.^1^ Despite these advantages, many hindrances have prevented peptide drugs from playing a major role in therapeutics, such as low membrane permeability, poor serum stability, and poor oral bioavailability.^2^ Initially developed peptide therapeutics were related to natural peptide hormones, such as insulin, oxytocin, vasopressin, and somatostatin.^3−7^ Primarily, peptide research in the mid- to late-twentieth century focused on different chemical modifications of these hormones for the development of peptide drugs with improved pharmacologic properties. Successful campaigns took advantage of incorporation of a variety of modifications to the peptides to prevent degradation from serum proteases or reduction of the disulfide bonds that were essential to the peptides.^8^ Recently, advances in synthesis, screening techniques, and delivery methods have allowed for peptides to become promising candidates to treat a wide variety of diseases, and over 52 peptide drugs have been approved in the past two decades.^2^ A recent drastic market hike of peptide therapeutics is due to the approval of GLP-1 agonists for weight loss. Driven by a huge market size surpassing 13 billion USD in 2023 and a projected annual growth rate of 4.8% (GLP-1 Receptor Agonist Global Market Report 2023, Reportlinker.com), there has been a tremendous burst of different biotech companies launched for the development of further GLP-1 analogs.^9−12^ Therapeutic peptides have now diversified into multiple classes, such as hormones,^13^ neurotransmitters,^14^ ligands,^15^ and anti-infective agents.^16^ One of the main advantages of peptides is the ability to easily screen large peptide libraries to identify therapeutic candidates. There are numerous methods to generate peptide libraries that range in diversity from 10^6^ to 10^15^ peptides, and several excellent reviews for them are available (see Figure 1 for an overview of these techniques and references to recent reviews).^17−20^ Of these, phage display has emerged as one of the most versatile and easily adaptable techniques over the past three decades. This review will focus on recent developments in techniques to integrate noncanonical amino acids (ncAAs) and other motifs into phage-displayed peptide libraries.

### 1.2 M13 Bacteriophage Structure

Prior to discussing modifications of peptides displayed on bacteriophages, it is important to understand the structure of the bacteriophage itself. While there has been some experimentation using other types of phages for phage display, the majority of studies have taken advantage of filamentous bacteriophage. As the name suggests, filamentous phages consist of long filaments that are approximately 1 μm long and 6 nm wide.^21^ They have been found to infect various genera of Gram-negative bacteria, with a few reports of filamentous phages infecting Gram-positive bacteria.^22^ Of the filamentous phages, the most well classified phages are those that infect using the F pilus of *Escherichia coli* and are known as f1, M13, or fd phages. M13 phages consist of viral proteins encapsulating a circular positive single-stranded DNA (+ssDNA) genome that encodes 9 open reading frames that produce 11 different proteins through the use of alternative start codons.^23^ In regard to phage display, an important aspect of M13 phage infections is that due to their slow replication and ejection from the cells, they do not lyse their hosts resulting in high phage titers (10^13^ phages/mL of culture).^22,24^ They also tolerate a wide variety of structural modifications,^25^ show high stability in a wide range of temperatures,^26^ and are very resistant to proteolysis.^27^ This makes them ideal for the display of peptide and protein libraries for selections against therapeutic targets.

**Figure 2:**
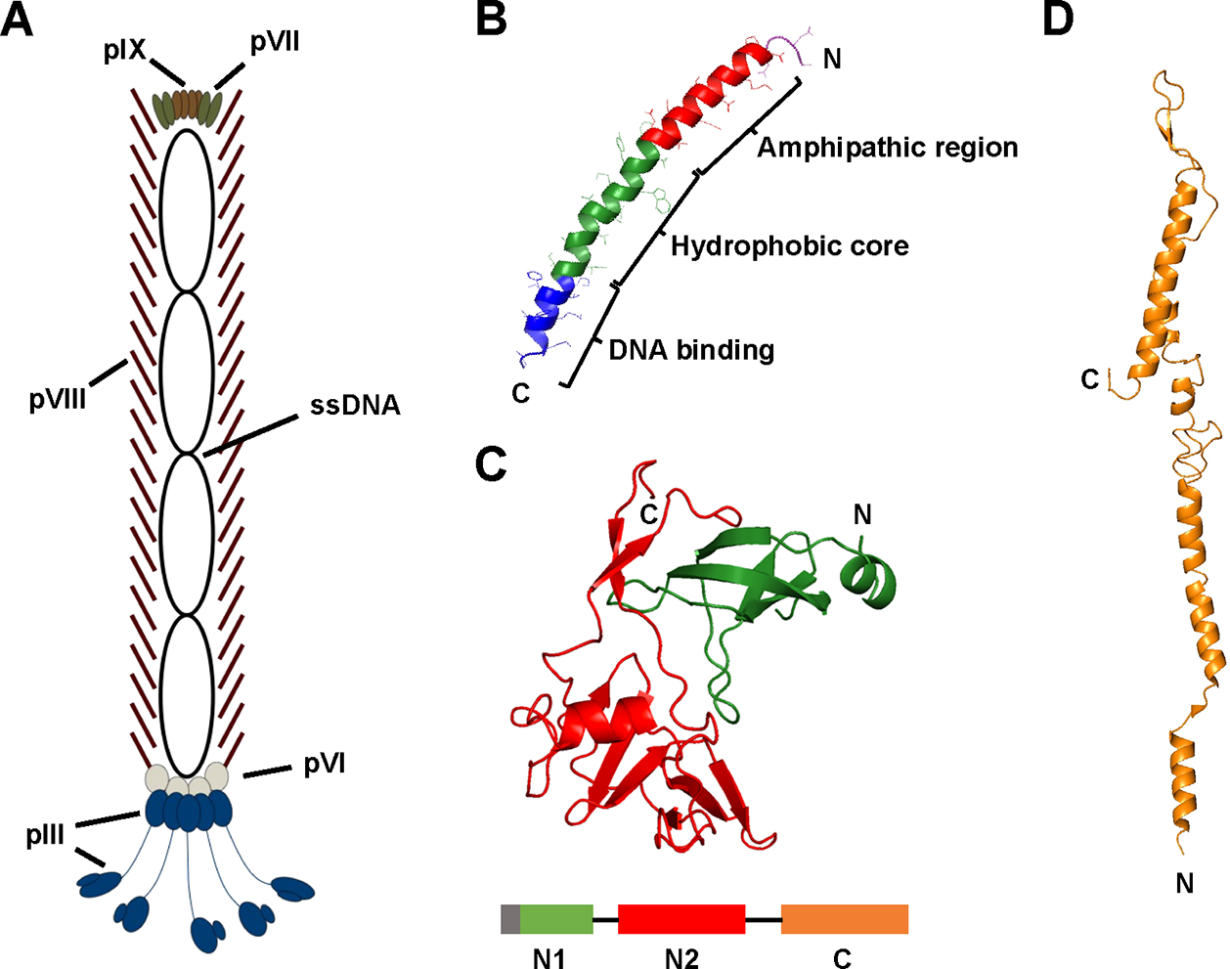
Structure of M13 bacteriophage. A) Representation of the M13 virion encapsulating its +ssDNA genome. B) Crystallography of pVIII (PDB: 2C0W) with colors assigned according to functionality of the region and the N- and C-termini labeled. Interactions between the hydrophobic core are responsible for maintaining structural integrity of the capsid. C) Structure of the pIII protein’s N1 (A19-P83, green) and N2 (E109-A235, red) domains (PDB: 1G3P) with a depiction of the full-length encoded protein below. The N1 domain contains a signaling peptide (gray) that is cleaved after incorporation in the membrane. Glycine-rich linkers are depicted as black lines between the three domains. N and C in the crystal structure refer to the N- and C-terminus. D) Cryo-EM of C-terminal domain (D275-K422) within the packaged virion (PDB: 8B3O) demonstrates a helical nature for insertion into the virion. The N- and C-termini in the structure are labeled as N and C.

The M13 bacteriophage virion consists of five types of coat proteins encapsulating a circular ssDNA genome (Figure 2A). In wild type M13 phages, the major coat protein is pVIII, with each virion containing roughly 2700 copies surrounding the circular ssDNA. A small, disordered N-terminal domain of pVIII consisting mainly of negatively charged residues is solvent exposed in the virion.^28^ The rest of the protein comprises an α helix that contains an amphipathic region (residues 5–20), hydrophobic region (residues 21–39), and then a positively charged region (residues 40–49) that interacts with the ssDNA (Figure 2B).^28^ The virion is held together through hydrophobic interactions between the alpha helices of adjacent pVIII proteins that spiral in a 5-fold symmetry around the encapsulated ssDNA.^29^ The phosphate backbone of the ssDNA interacts with the positively charged region of pVIII, and the length of the virion is typically dependent upon the length of ssDNA being packaged.^22^

The virion is then capped on either end by a variety of proteins, with pVII and pIX on the “head” and pIII and pVI on the “feet” of the phage. pVII and pIX consist of about 30 residues each and are the initial proteins (approximately five of each protein) excreted from the cell during packaging.^30^ There is much less structural information known about these proteins, but it is thought that C-terminal ends recognize the packaging signal in the phage genome to initiate assembly.^31^ Phage assembly is terminated by the addition of pVI and pIII to the virion. While the interactions between pIII and pVI are not well characterized, there are five copies of each protein, and it is proposed that they maintain the 5-fold axial symmetry that pVIII displays.^22^ pVI is a moderately sized protein consisting of 112 residues that are mainly hydrophobic and is predicted to have three transmembrane alpha helices.^22,30^ pIII is significantly larger than the other proteins in the virion and is composed of three different domains (N1, N2, C) that are joined by glycine-rich linkers, along with an N-terminal signaling peptide for localization to the membrane (Figure 2C). N1 and N2 are similarly folded with each containing two antiparallel β-sheets and an α-helix.^32,33^ Infection into host cells is mediated through the N1 and N2 domains of pIII, while the C domain appears to be involved with interactions to pVI to terminate viral assembly.^22^ Through recent Cryo-EM studies, it was determined that the C-terminal domain is primarily composed of alpha helices for insertion within the virion (Figure 2D).^34^ The C-terminal end of the protein also contains a transmembrane region that serves to anchor the protein in the host membrane during assembly.^35^

### 1.3 Overview of Phage Display Biopanning

**Figure 3:**
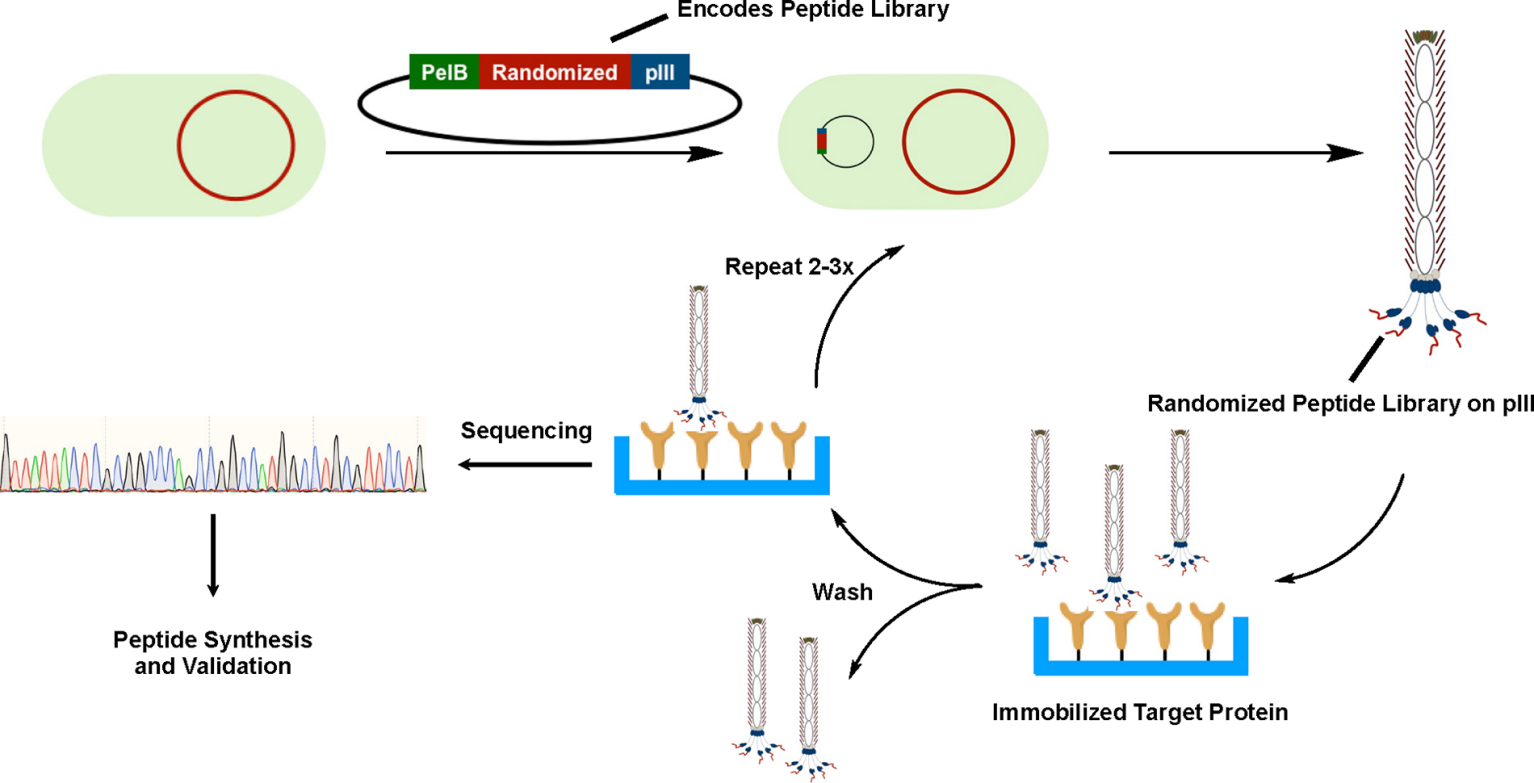
Overview of phage display biopanning. Phage-displayed peptide libraries are typically produced using *E. coli* that are transformed with two plasmids. One plasmid produces a modified phage protein that has a genetically encoded peptide library fused to it, while the other plasmid provides all other necessary phage proteins. These phages are then used to bind to an immobilized target, protein or other biomolecule, to allow for washing of the phages that do not bind. After elution, phages are amplified again in *E. coli* to repeat the selection until enrichment of peptides in the phage library is observed through sequencing of the phage DNA. After sequencing, the corresponding peptides are synthesized and validated for binding to the target of interest.

Over the past 40 years, researchers have taken advantage of M13 bacteriophages to genetically encode large peptide and protein libraries. These libraries are then used to select for peptides or proteins to bind to desirable targets through iterative rounds of selection (Figure 3). The packaging mechanism can tolerate large insertions and deletions of the phage ssDNA, as long as the packaging signal and origins of replication remain intact. Thus, modifications to the phage genome through molecular biology techniques allow for the insertion of large, randomized regions into the genome that can be subsequently expressed as a fusion to other phage proteins. Although all phage coat proteins have been exploited to display libraries, the most commonly used proteins for display are pVIII and pIII.^22^ Displayed peptide sequences usually are spliced into the phage protein sequences just after the signaling peptide domain to ensure that the fusions are incorporated into the membrane prior to virion formation. Peptides displayed using pVIII typically modify the N-terminal end of the protein, which is exterior to the phage virion. Insertions of less than seven amino acids to pVIII is tolerated for virion formation, however, larger libraries necessitate coexpression of wild-type pVIII to generate functional virions.^36^ Also, when displaying peptides on pVIII one should be aware of potential valency effects, as there are more than 2700 copies per virion. Thus, pIII display has been the preferred method for many different applications.

There are three different regions that can be exploited to display peptide libraries on pIII: the N-terminus, between the N2 and C-terminal domain, and on the C-terminus. Initially, fusion of peptides to the middle region (between the N2 and C domains) of pIII was explored to avoid adversely affecting phage infectivity.^37^ While the display of peptides in this location was possible, there was a decrease in infectivity with these clones. Therefore, another system that encoded the peptide library fused to the N-terminus of pIII was subsequently developed.^38^ Similar to the pVIII fusions, large peptides displayed on the N-terminal domain of pIII can diminish infectivity of the phages and the coexpression of wild-type pIII is necessary alongside these proteins. Alternatively, methods have been developed to cleave the peptide fusion off with trypsin prior to infecting cells, which allows for the expression of large proteins on the N-terminus of pIII.^39^ Alongside this system, expression of peptides on the C-terminus of pIII has also been explored and proven successful, although this is typically less popular and has been reserved for peptides that require special processing by cytoplasmic enzymes.^40^ It is worth noting that there are different systems for producing libraries on pIII that are characterized by the location of library insertion. The first developed system, Type 3 display, directly modified the pIII gene in the M13 phage genome to afford a modified pIII with the fused peptide library. This results in every pIII copy containing the modified peptide fusion. However, to circumvent the possibility of the peptide fusion hindering infectivity, other systems that provide wild-type pIII protein have been developed.^25^ Type 33 display refers to the incorporation of genes encoding both wild-type pIII and the peptide-fusion pIII into the phage genome. Therefore, a mixture of modified and wild-type pIII proteins are displayed on the surface of the virion and infectivity is not reduced for these variants. One major disadvantage of Type 3 and Type 33 display is that they require insertion of the peptide library directly into the phage genome. This can result in the amplification and enrichment of phages that acquire mutations in proteins other than the pIII-library fusion, severely hindering the potential for selections using these libraries.^41,42^

One way to avoid this undesired enrichment of parasitic phage genomes is to split the phage production into two different plasmids. In Type 3+3 display, a phagemid is used that encodes the peptide-fusion pIII and contains the f1 origin for packaging of the virion. Alongside this, a helper phage genome is also used that encodes for all phage proteins but has a split f1 origin to favor packaging of the phagemid DNA over the helper phage genome.^43,44^ To select for the presence of each plasmid, they both contain antibiotic resistance casettes, typically for kanamycin (helper phage) and ampicillin (phagemid).^45^ This system affords less risk of accumulating mutations within non-pIII related proteins in comparison with Type 3 and Type 33 systems. Also, having the library within the phagemid results in numerous advantages including improved pIII expression, increased transformation efficiencies, facile cloning, and inducible promoters.^45^ However, because the 3+3 system expresses wild-type pIII alongside the modified version, slowly expressing pIII-fusions can have low coverage in the packaged virions.^46^ To circumvent this, Type 3+0 systems have also been developed, where the helper phage genome produces either no pIII or a dysfunctional pIII.^39,46−48^ Similar to the Type 3 system, this affords virions where all pIII subunits are modified in the virion. For large libraries that are produced using the Type 3+0 system, functionality of pIII may be reduced, so it is generally required to cleave the modification to pIII prior to infection of cells.^46^ In this review, most of the systems discussed take advantage of the phagemid-based systems (Type 3+3 or Type 3+0) for expression, although there are a few reports that directly modify pIII on the phage genome.

### 1.4 Review Outline

Many works have been published that use phage display or other peptide display techniques to identify therapeutic peptides. In this review, we will focus on recent developments for phage-assisted identification of peptides that contain ncAAs and other noncanonical motifs, with a particular focus on consolidating methods to generate modifications on phage. Table 1 summarizes recent reviews on additional topics that are relevant to this work, including overviews of phage display,^25,49,50^ genetic code expansion,^51−54^ platforms for genetically encoded libraries,^55,56^ and peptide therapeutics.^8,57−59^ This review begins with the application of genetic code expansion to phage-displayed libraries (Section 2), followed by chemical post-translational modifications (Section 3), and it concludes with a general overview of remaining challenges in displaying noncanonical motifs on phages (Section 4).

**Table 1:** Summary of Recent Relevant Reviews on Peptide Libraries, Genetic Code Expansion, and Peptide Therapeutics

Topic	Publication Title	Year	Reference
Historical Accounts of Phage Display	Phage Display	1997	(25)
	Phage Display: Simple Evolution in a Petri Dish (Nobel Lecture)	2019	(49)
	Harnessing Evolution to Make Medicines (Nobel Lecture)	2019	(50)
Genetic Code Expansion	Expanding and Reprogramming the Genetic Code	2017	(51)
	Reprogramming the Genetic Code	2021	(52)
	Pyrrolysyl-tRNA Synthetase: An Ordinary Enzyme but an Outstanding Genetic Code Expansion Tool	2014	(53)
	Update of the Pyrrolysyl-tRNA Synthetase/tRNAPyl Pair and Derivatives for Genetic Code Expansion	2023	(54)
Genetically Encoded Libraries	Methods for generating and screening libraries of genetically encoded cyclic peptides in drug discovery	2020	(55)
	Expanding the Chemical Diversity of Genetically Encoded Libraries	2020	(56)
Cyclic Peptides	Phage Selection of Cyclic Peptides for Application in Research and Drug Development	2017	(57)
	Macrocyclic Peptides as Drug Candidates: Recent Progress and Remaining Challenges	2019	(58)
Peptide Therapeutics	Peptide-based drug discovery: Current status and recent advances	2023	(59)
	Trends in Peptide Drug Discovery	2021	(8)

## 2 Applications of Genetic Code Expansion to Phage Displayed Peptides

### 2.1 Overview of Orthogonal Genetic Code Expansion

In nature, there are typically only 20 canonical amino acids (cAAs) that are incorporated into proteins through translation of the genetic code. This provides significant limitations to the chemical diversity that can be encoded in phage libraries. Twenty cAAs are classified into several categories based on their side chains as positively or negatively charged, polar, hydrophobic including both small aliphatic and large aromatic, and special cases including cysteine, glycine and proline. Chemical groups that are typically observed in approved small molecule drugs such as halides, nitro, alkyne, and nonimidazole heteroaromatic rings are missing in cAAs. However, studies over the past 50 years have looked to expand the genetic code to allow for the ability to generate proteins and peptides containing novel moieties with interesting chemistries.

Genetic code expansion by amber suppression was pioneered by several laboratories in the 1970s and later expanded by the Schultz Lab in the 1990s, where chemically synthesized tRNAs were used to recognize and incorporate an ncAA at the amber stop codon (UAG) site in an mRNA.^60^ An important advantage is that translation is stopped when there is no incorporation at the amber codon, thus preventing background proteins from being expressed. Although being limited to *in vitro* systems, this technique has since been generalized to encode for a wide variety of amino acids by taking advantage of flexizymes, or RNA molecules that serve as catalysts for tRNA acylation.^61^ Applications of flexizymes to peptide display have recently been reviewed thoroughly, and any readers interested in this should be directed to the review by Suga et al.^18^ Nevertheless, limitations still exist with *in vitro* suppression of amber codons, especially considering proteins and systems that require cellular environments for processing, such as with phage display.

Further work by Peter Schultz and others led to the ability for *in vivo* genetic code expansion in *E. coli*, greatly expanding the scope of amber suppression techniques. For *in vivo* translation to be successful, several key components must be present in the system.^62^ The ncAA needs to be able to be transported into the cell, but it cannot be recognized by endogenous translational machinery. Also, the tRNA/tRNA synthetase pair for the ncAA must be orthogonal to the endogenous system so that no cAAs are incorporated at the desired site and no ncAAs are incorporated at undesired codons. Interestingly, a *Methanococcus jannaschii* tRNA^Tyr^/tyrosyl-tRNA synthetase pair (MjTyrRS) was identified and evolved to orthogonally suppress amber codons in *E. coli* with high selectivity for ncAAs.^62^ This allowed for adaptable *in vivo* translation of ncAAs in *E. coli* and also presented a system to evolve novel tRNA/tRNA synthetase pairs for new ncAAs. Other tRNA/tRNA synthetase systems that were explored for genetic code expansion in *E. coli* include *Pyrococcus horikoshii* tRNA^Lys^/lysyl-tRNA synthetase and even *E. coli* tRNA^Trp^/tryptophanyl-tRNA synthetase by swapping them with *Saccharomyces cerevisiae* tRNA^Trp^/tryptophanyl-tRNA synthetase.^63,64^

**Figure 4:**
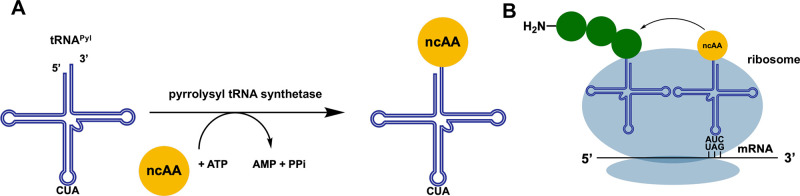
Genetic code expansion using pyrrolysyl tRNA synthetase. A) In amber suppression, pyrrolysyl tRNA synthetase (PylRS), wild type or evolved, charges its cognate tRNA (tRNA^Pyl^) with an ncAA. PylRS and its mutants have been evolved to recognize more than 200 ncAAs to charge tRNA^Pyl^. B) The ncAA on the charged tRNA^Pyl^ is then incorporated into a peptide at the ribosome by suppression of an amber (UAG) codon on the corresponding mRNA.

Shortly after using tRNA^Tyr^/MjTyrRS as an artificial system for the genetic encoding of ncAAs, a naturally occurring tRNA/synthetase pair was discovered in *Methanosarcina barkeri* and other *Methanosarcina* species for encoding pyrrolysine (Pyl) at the amber codon.^65−67^ It was found that the tRNA synthetase (Pyrrolysyl-tRNA synthetase or PylRS) recognized a unique tRNA (tRNA^Pyl^) gene (PylT) that had a CUA anticodon (Figure 4).^68,69^ PylRS contains an N-terminal domain that serves to recognize tRNA^Pyl^ and a C-terminal domain that catalyzes the charging of tRNA^Pyl^ with Pyl. The original PylRS demonstrated promiscuous substrate selectivity and was found to charge tRNA^Pyl^ with and therefore encode for a variety of ncAAs that were pyrrolysine analogs or structurally distinct derivatives.^70−72^ This promiscuity is aided by both the lack of an editing domain and that the interactions between PylRS and Pyl are primarily driven through hydrophobic interactions. Also, PylRS has been shown to have promiscuity to even incorporate α-hydroxy derivatives of α-amino acids due to its low interactions with the α-amine of Pyl.^73,74^ Further modifications to PylRS have allowed for increased promiscuity and diversity of ncAAs that can be encoded using the tRNA^Pyl^/PylRS pair, with currently hundreds of different ncAAs able to be incorporated.^53^ Due to its unique structures, tRNA^Pyl^/PylRS is largely orthogonal to many commonly used lab cells including *E. coli*, *S. cerevisiae*, and mammalian cells, enabling easy transfer of evolved tRNA^Pyl^/PylRS pairs between different cellular systems. For the aforementioned reasons, tRNA^Pyl^/PylRS has become the dominant system for the genetic code expansion.

**Figure 5:**
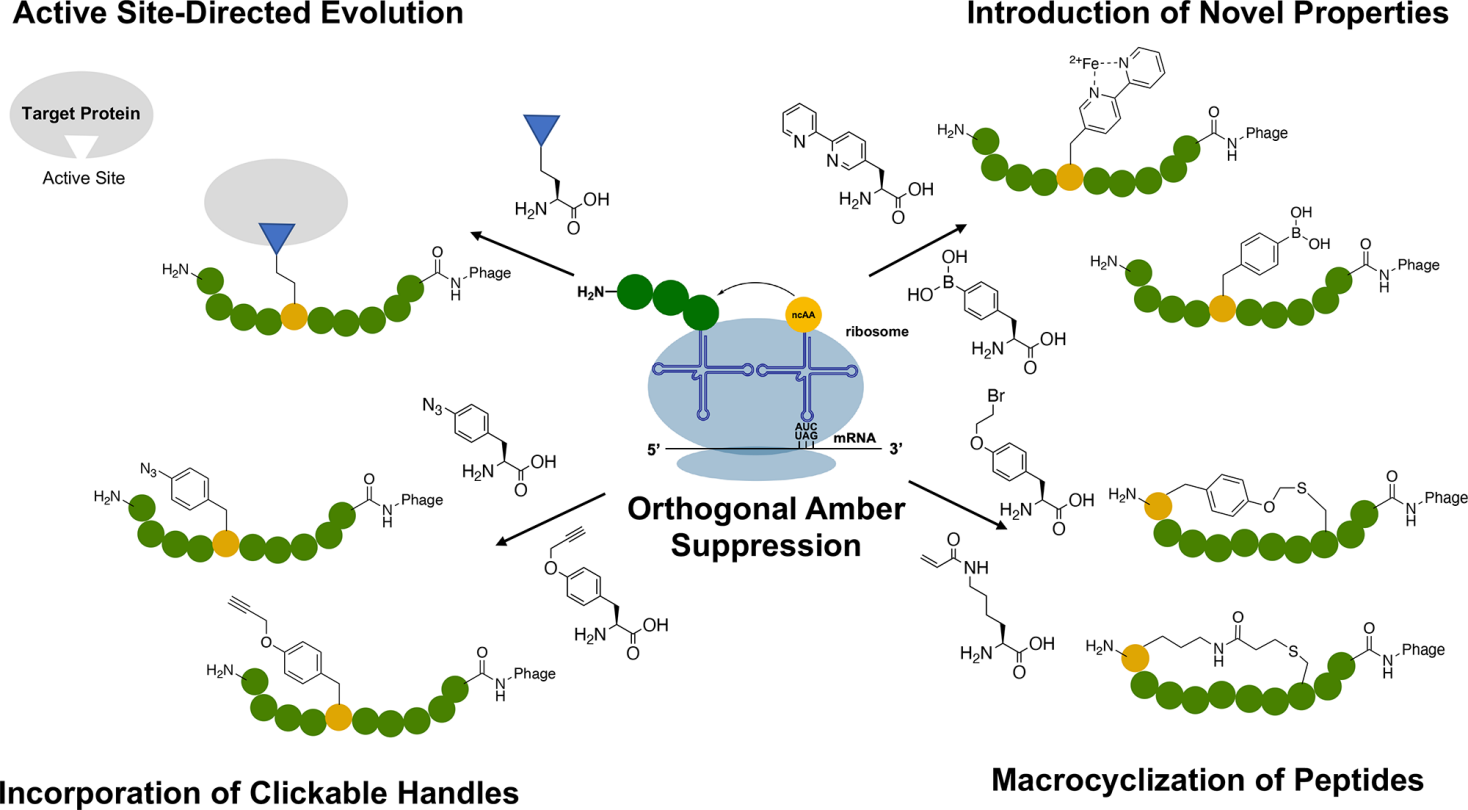
Overview of ncAA incorporation into phage libraries. A variety of advantageous moieties have been installed into phage libraries through genetic code expansion via amber suppression. These include the ability to direct selections toward active sites by incorporation of ligands into ncAAs, introduction of metal-chelating and reversibly covalent ncAAs, proximity-driven macrocyclization with cysteines, and introduction of clickable handles into phage libraries.

Although there have been elegant systems developed to evolve substrates for tRNA-synthetases using fluorescence-based sorting^75^ and phage-assisted continuous evolution,^76^ inherent limitations in ncAA diversity still exist. All evolution systems for orthogonal translation require the ncAA-tRNA complex to be a substrate of the ribosome, which prevents many different amino acids from being efficiently incorporated into peptide chains. While the ribosome can tolerate incorporation of α-hydroxy derivatives of α-amino acids and *N*-methyl amino acids, α,α-disubstituted amino acids, β-amino acids, and d-amino acids are poor substrates for translation.^77^ Additional backbone modifications, such as *N*-alkylation, can also hinder efficient translation at the ribosome.^78^ Alongside modifications to the backbone, there are limitations to the side chains as well when using orthogonal translation systems. For example, tRNAs loaded with negatively charged amino acids have lower affinities for elongation factor Tu (EF-Tu), which initially hindered the development of a system to incorporate phosphoserine.^79,80^ Large side chains may also prevent binding to the tRNA synthetase and/or ribosomal pocket. In spite of these limitations, there is still a wide variety of amino acids that have been efficiently encoded into proteins using amber suppression systems. In the past ten years, researchers have also begun adapting the versatility of the genetic code expansion technique to phage display and have taken advantage of it in a variety of applications. A general overview of these applications is given in Figure 5.

### 2.2 Initial Studies for ncAA Incorporation into Phage Displayed Proteins

The first reported genetically encoded ncAA incorporated into phage libraries was selenocysteine (Sec), where Sandman et al. took advantage of the endogenously expressed *selABCD* operon to incorporate Sec at an opal codon (Table 2).^81^ In this study, phages were successfully isolated that were dependent upon Sec for incorporation by integrating the selenocysteine insertion sequence (SECIS) and an opal-containing library upstream of gIII in the phage genome. While the incorporation of Sec gives access to novel reactivities, using the endogenous expression system limits the ability to encode other ncAAs. Shortly after this landmark study, Tian et al. significantly increased the diversity of ncAAs able to be displayed in phage libraries by taking advantage of mutant MjTyrRS/tRNA^Tyr^ pairs to orthogonally translate five different ncAAs into phage-displayed peptides (MeY, AzF, AcF, BpF, and NpA; Table 2).^82^ In this study, they were able to demonstrate phage expression that was dependent on ncAA incorporation by fusing an amber-containing streptavidin binding peptide (SBP) to the N-terminal domain of pIII. Several different ncAAs that were used could be incorporated at amber codons through specifically engineered tRNA synthetases based on the original MjTyrRS, and each system is described in Table 2.^62,83−86^ One interesting application from this work was the ability to incorporate *p*-azido-phenylalanine (AzF, Table 2) into phage proteins, which allows for potentially clickable modifications to phage libraries (Figure 5).^82^

**Table 2:**
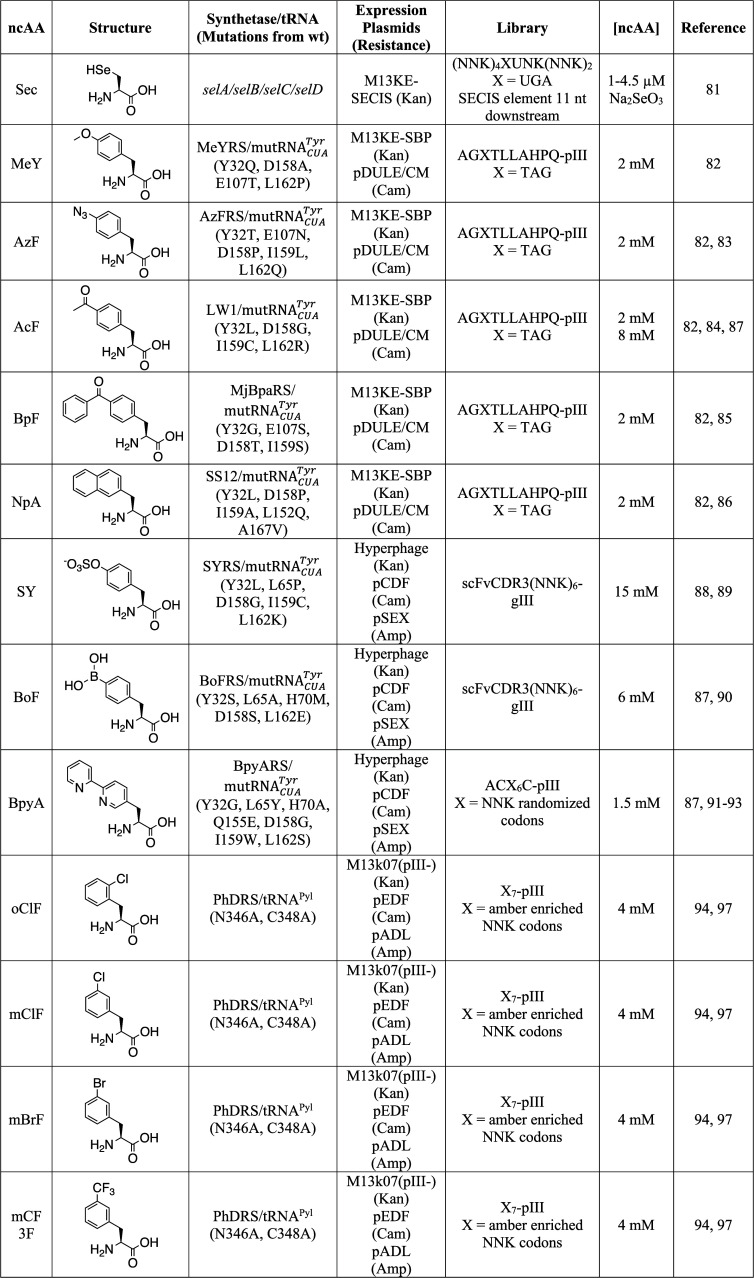

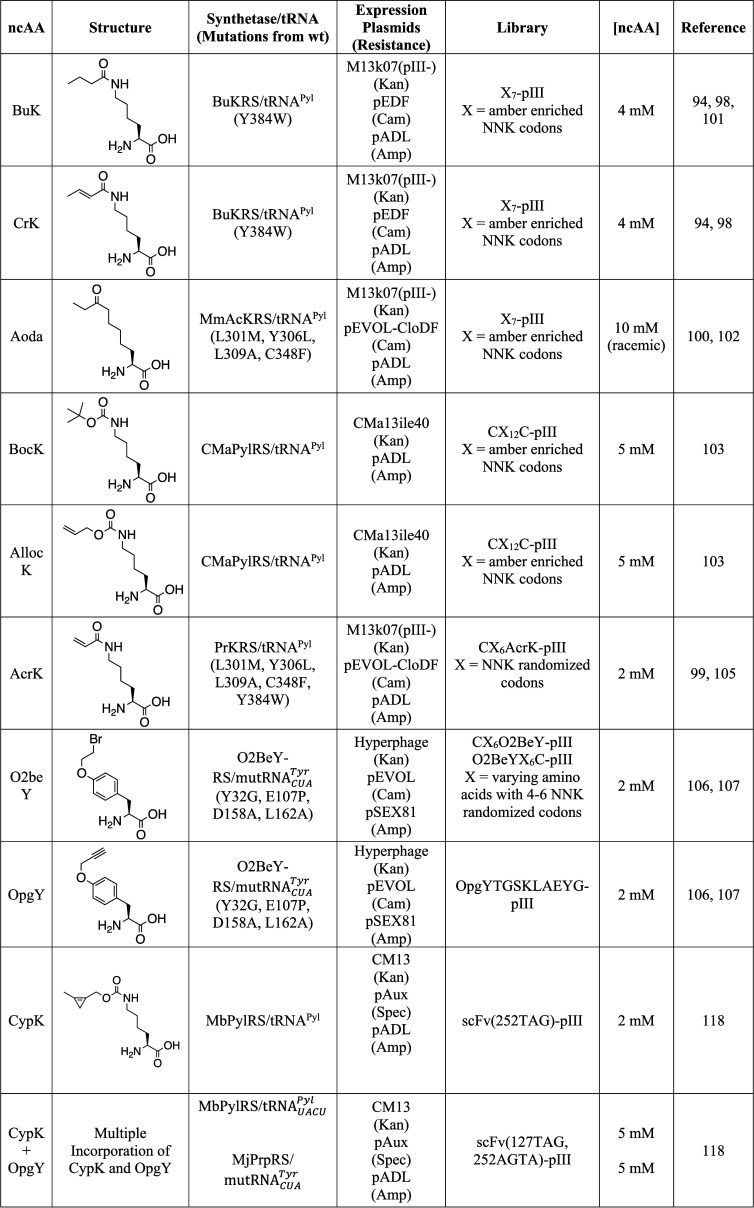
Genetic Incorporation of ncAAs into Phage Libraries

Despite successful ncAA integration into phage-displayed libraries in the early 2000s, the application of these libraries to identify ligands for certain targets has been limited due to the inability to outcompete production of wild-type phage that do not contain amber codons within their genome. Although amber suppression systems are optimized to efficiently encode ncAAs, they still produce less protein than those containing strictly sense codons due to competition with termination machinery. This results in lower pIII production, and subsequently lower phage yields for libraries containing ncAAs. Thus, any phage libraries that contained a mixture of sense-only and amber-containing clones would be heavily biased toward the sense-only clones. One innovation that helped to counter this was the development of hyperphages, helper phages that contain a nearly complete deletion of gIII in their genome, that gave the ability to create large phagemid libraries completely dependent upon amber suppression for phage production.^46^ Additionally, the Schultz Lab demonstrated that the bias for sense-only codons could be lessened by increasing pIII production through expressing phages with higher concentrations of ncAA and prolonged expression times at lower temperatures (30 °C).^87^ These discoveries allowed for the generation of a sulfotyrosine (SY, Table 2) containing antibody library through genetic code expansion using an MjTyrRS mutant to incorporate SY into amber codons of a randomized CDR3 region and subsequent identification of SY containing scFv antibodies that selectively bound to gp120.^87,88^ Similarly, introduction of boronic acids (BoF, Table 2) into a phage-displayed scFv library by using a mutant MjTyrRS afforded the ability to identify proteins that potently bind geminal diols and gave the possibility to develop antibodies that interact with glycans.^89,90^ Other interesting examples of ncAA incorporation into phage libraries involved using an MjTyrRS mutant to incorporate bipyridyl-alanine (BpyF, Table 2) into a randomized disulfide-cyclized peptide library of six amino acids and also into zinc-finger peptides.^91−93^ In doing this, they identified peptide ligands that bound to Ni^2+^ with sub micromolar affinity (Table S1, Peptides 1 and 2) and also developed novel iron(II)-finger proteins.^92,93^ Follow-up studies with these libraries may allow for the evolution of metallopeptide catalysts and other beneficial probes that take advantage of metals for activity. While these initial cases all demonstrate the ability and potential of genetic code expansion in phage libraries, they were reliant on incorporation of already strong ligands into the ncAAs for successful selections. Until recently, phage-assisted identification of *de novo* peptides that contained ncAAs with modest affinities for targets was hindered by parasitic peptide sequences that lacked amber codons and outcompeted ncAA-containing libraries.

### 2.3 Development and Application of Amber-Obligate Peptide Libraries

**Figure 6:**
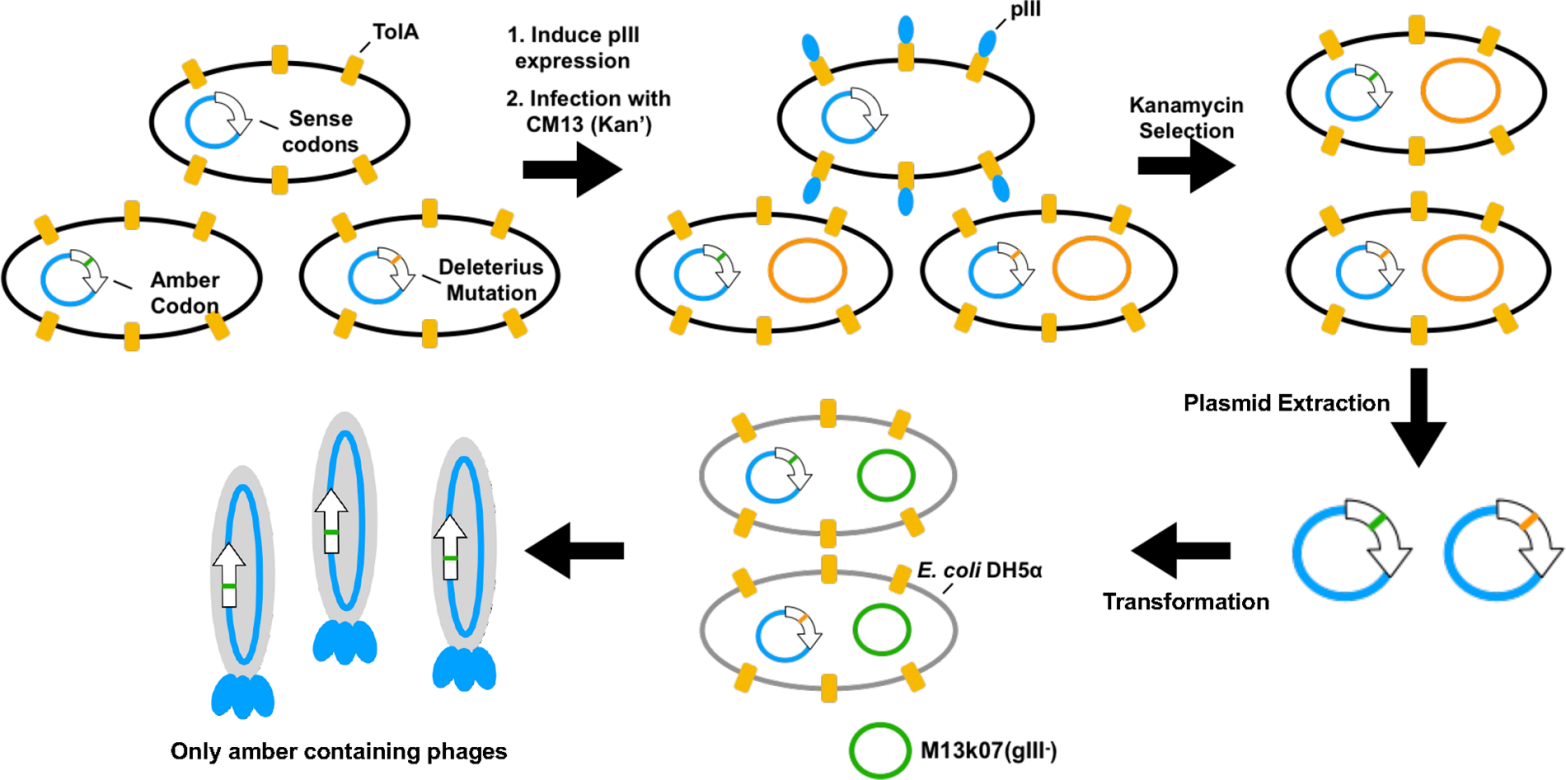
Generation of amber obligate phage libraries through superinfection immunity selection. Plasmids expressing pIII-library fusions are induced for expression without amber suppressing mechanisms, resulting in sense-only clones expressing pIII and preventing infection by a helper phage CM13. Cells are then selected for kanamycin resistance and plasmids extracted, isolating the clones that lack pIII expression. After transformation and phage expression in amber suppressing cells (DH5α), amber obligate libraries are generated.

Although amber suppression techniques were first applied to phage-displayed libraries in the early 2000s, there were only a handful of examples of them successfully employed in selections in the following decade. One of the main complications was due to the inability to isolate amber-containing phage libraries from parasitic sense-only libraries. In 2020, Tharp et al. reported a strategy to enrich for amber-containing peptides *in vivo* that took advantage of superinfection immunity of filamentous phages (Figure 6).^94^ Because expression of pIII results in interactions with the TolA receptor, any cells that express pIII cannot be infected by other filamentous phages that are dependent upon TolA for entry.^95,96^ This allows for the ability to isolate libraries that contain amber codons by initiating pIII expression in the absence of amber suppression machinery and subsequent infection with a helper phage to give *E. coli* with the amber-containing libraries kanamycin resistance.^94^ All other libraries (sense-only) that express pIII can then be eliminated through selection with kanamycin.^94^ This differentiation of amber-containing and sense-only clones affords the ability to enrich amber codons at any stage in a phage selection. Thus, ncAAs that give modest affinity to protein targets can be used without concern for inadvertently enriching parasitic sense-only clones. Additionally, the amber enrichment technique facilitates the generation of libraries that contain randomly positioned amber codons, rather than it being fixed in one position. This gives increased diversity of phage libraries and higher potential in identifying potent ligands.

Tharp et al. took advantage of the newly developed amber-obligate library to successfully incorporate six novel ncAAs into phage libraries.^94^ Four phenylalanine derivatives (oClF, mClF, mBrF, and mCF3F, Table 2) and two lysine derivatives (BuK and CrK, Table 2) were incorporated using the previously developed PhdRS and BuKRS mutants of PylRS, respectively.^97−99^ Initial selections against TEV protease and streptavidin using these amber-obligate phage libraries and the phenylalanine derivatives demonstrated the robust ability to isolate ncAA-containing peptide ligands for proteins even in the absence of a strong warhead to direct binding (Table S1, Peptides 3–6).^94^ In addition to this, they also demonstrated a phage-assisted active site-directed ligand evolution (PADLE) technique (Figure 5), with BuK directing binding of the peptide library toward the active site of sirtuin 2, which ultimately resulted in low nanomolar inhibitors of the protein (Table S1, Peptides 7 and 8).^94^ This technique shows promise especially in discovering ligands/inhibitors for proteins that recognize post-translational modifications. PADLE has recently been applied to different epigenetic readers, including histone deacetylase 8 (HDAC8)^100^ and ENL-YEATS.^101^ For the development of HDAC8 inhibitors, an acetyl-lysine analog (Aoda, Table 2) was incorporated into phage libraries using a previously developed AcKRS mutant of PylRS and subsequently used to develop sub nanomolar isoform-selective inhibitors (Table S1, Peptide 9).^100,102^ In the application of PADLE to develop ENL inhibitors, BuK-containing phage were panned against the ENL YEATS domain and low nanomolar inhibitors of the protein’s interaction with an acetylated peptide were discovered (Table S1, Peptides 10 and 11).^101^ Given the success in identifying ligands for multiple epigenetic readers, we envision PADLE can be applied to a wide variety of proteins that recognize post-translational modifications for the identification of selective probes and inhibitors.

While the development of amber-obligate libraries was a revolutionary technique for ncAA incorporation in phage libraries, there were still a few aspects of the system that hindered its application. The first of these is that the amber enrichment procedure was tedious and required multiple rounds of amplification/electroporation. This process magnifies the limitation of electroporation on library size and may introduce biases in the libraries themselves. Also, the use of a three-plasmid expression system appeared to give lower phage yields in comparison to two-plasmid systems. To combat these issues, a novel amber-encoding helper phage, CMa13ile40, has been recently developed that both shortens the amber enrichment process and results in higher phage yields.^103^ CMa13ile40 was developed by insertion of a *Methanomethylophilus alvus* PylRS/tRNA^Pyl^ cassette into the intergenic region of helper phage CM13d3. In addition to shortening the amber enrichment process, the new helper phage also demonstrated higher specificity for packaging of the phagemid-containing library in comparison to commercially available helper phages.^103^ In this work, two additional ncAAs were incorporated into phage libraries, BocK and AllocK (Table 2), and the influence of different ncAAs on selection was demonstrated with the isolation of peptides with low micromolar affinity for the membrane-bound E3 ligase ZNRF3 (Table S1, Peptides 12–14). Although the use of CMa13ile40 significantly shortens the amber enrichment process, development of new helper phage derivatives for incorporation of other amino acids will be necessary for expanded applications.

### 2.4 Genetically Encoded Macrocyclic Peptide Libraries

In addition to integrating ncAAs within phage-displayed peptide libraries to direct selections, there have been a few studies that have taken advantage of ncAAs to generate genetically encoded cyclic peptide libraries (Figure 5). Interest in applying cyclic peptides as therapeutics has risen recently due to increased stability and potency in comparison to their linear counterparts.^104^ Typically, phage-displayed peptide libraries are linear, resulting in limitations to their application for therapeutic development. Therefore, incorporation of ncAAs that have intrinsic reactivities to catalyze cyclization of peptides on the displayed libraries gives great potential for identifying therapeutic lead compounds. One challenge in the incorporation of reactive ncAAs is the fine-tuning of the reactivity of the ncAA—it must remain unreactive until incorporation into phage libraries and then react with the incorporated peptides. If the ncAA is too reactive, it will be modified before incorporation into libraries and have difficulty being encoded. The first study that successfully used an ncAA to cyclize peptide libraries on phage took advantage of an acryloyl-lysine (AcrK, Table 2) to react with an N-terminal cysteine flanking a peptide library.^105^ The AcrK was genetically encoded using a mutant PylRS (PrKRS) that had been previously evolved from AcKRS.^99^ Biotin pulldown assays indicated approximately 60% efficiency in displaying cyclic peptide libraries and that the cyclization was dependent upon the N-terminal cysteine.^105^ The cyclic peptide library was subsequently used to identify cyclic peptide inhibitors for TEV protease and HDAC8, with each selection resulting in peptides with low micromolar affinity for each protein (Table S1, Peptides 15–17).^105^

Shortly after the development of phage-displayed cyclic peptide libraries using AcrK, an additional strategy (MOrPH-PhD) was reported that took advantage of a bromoethyl-tyrosine derivative (O2beY, Table 2) to cyclize a variety of cysteine-containing phage libraries on pIII.^106^ Prior to incorporation of O2beY into phage, in a separate study Fasan et al. developed an MjTyrRS mutant for O2beY (O2beY-RS) and rigorously characterized its ability to cyclize peptides containing cysteine at different positions relative to O2beY.^107^ They observed high cyclization efficiencies containing cysteine in the *i* + 2 to *i* + 8 position and also showed reactivity in the *i* – 6 and *i* – 8 positions.^107^ This prior validation gave them confidence in producing macrocyclic peptide phage libraries with the cysteines and O2beY at different positions. Interestingly, they observed a dependence in selection efficiency on the library type, as different proteins gave preference to libraries containing cysteine upstream or downstream to the ncAA (Table S1, Peptides 18–33).^106^ Ultimately, this study was afforded low nanomolar macrocyclic peptide ligands for The Keap1 Kelch Domain (Table S1, Peptides 28 and 29), and low micromolar ligands for the Sonic Hedgehog protein (Table S1, Peptides 32 and 33). These selections favored use of libraries with internal cysteines and an N-terminal O2beY, whereas the selections against streptavidin isolated proteins with O2beY on the C-terminal end (Table S1, Peptides 18–33). This highlights the necessity to create and screen diverse cyclic peptide scaffolds for the development of potent ligands for proteins. In addition to incorporation of O2beY, the same study also incorporated propargyl-tyrosine (OpgY, Table 2) at high efficiency in phage using the developed O2beY-RS.^106^ Although they did not demonstrate it, this amino acid could potentially be used to generate phage-displayed libraries labeled with pharmacophores or other therapeutically relevant moieties. All-in-all, these two studies highlight the use of ncAAs to genetically encode cyclic peptides on phage and should encourage further adaptions to target other proteins.

### 2.5 Outlook of Genetic Code Expansion in Phage Display

There have been numerous studies demonstrating the ability to increase the chemical diversity of phage-displayed peptide libraries since its inception in the early 2000s. In spite of the great versatility in amber suppression, there is still a need for improved systems, especially due to the poor stability of the N-terminal domain of PylRS.^108,109^ Ongoing efforts to improve upon the solubility and stability of the N-terminal domain include directed evolution,^109,110^ formation of chimeric interspecies PylRSs,^108,111,112^ and phage-assisted continuous evolution.^76,113^ In addition to developing novel systems for stabilizing PylRS and tRNA synthetase activities, investigation into new techniques to evolve for the encoding of novel backbones, large side chains, and charged side chains will help overcome current limitations on ncAAs that can be incorporated in orthogonal translation (see Section 2.1 for limitations in ncAA scope). A recent study by Schepartz et al. demonstrated the ability to expand substrate scope to encode for a variety of backbone modifications, such as α-hydroxy, α-thio, and *N*-formyl amino acids.^114^ Also, Chin et al. have devloped a novel tRNA display system to identify aminoacyl tRNA synthetases for β-amino acids, α,α-disubstituted-amino acids, and β-hydroxy acids.^115^ Subsequent integration of these suppression systems into phage display will allow for increased diversity of the displayed peptides and enhanced stability of the identified peptide ligands. Even with the development of highly efficient amber suppression systems, there is still a limitation to only incorporating one ncAA into the phage libraries and other systems will need to be employed to compete with the ability of *in vitro* translated mRNA displayed libraries that can simultaneously encode multiple ncAAs. Using two orthogonal tRNA/tRNA synthetase pairs in *E. coli*, the genetic incorporation of two different ncAAs simultaneously in one protein were demonstrated a decade ago.^116,117^ One can potentially use these cellular systems for the production of phages with multiple different ncAAs, which has been recently demonstrated. One report by Chin et al. for encoding multiple ncAAs into phage took advantage of both amber suppression and quadruplet codons to incorporate a propargyl-tyrosine (OpgY, Table 2) and cyclo-propene containing amino acid (CypK, Table 2), respectively.^118^ To increase amber suppression and allow for use in reading the quadruplet codon, they employed use of an orthogonal ribosome (riboQ1) that they had previously developed.^117^ When using riboQ1, they demonstrated enhanced amber suppression and were able to use a helper phage that contained wild-type pIII (CM13) to allow for monovalent antibody expression.^118^ Coupled with two different orthogonal tRNA synthetase mutants, they were able to achieve double incorporation of CypK and OpgY into an scFv-displayed phage, then demonstrated the ability to label both ncAAs using click reactions.^118^ This may be a promising system to incorporate multiple ncAAs into phage libraries and afford more diverse peptides. Further diversification of phage libraries using orthogonal translation will require the development of more techniques for genetic code expansion. The Chin Lab has also reported orthogonal translation of three different ncAAs, albeit not applied to phage libraries.^119^ Additional diversification may require adapting phage display to *E. coli* containing synthetic genomes, such as Syn61Δ3, to employ use of additional codons other than the amber codon.^120,121^

## 3 Chemical Post-translational Modifications of Phage-Displayed Peptides

### 3.1 Introduction and Initial Studies for Chemical Diversification of Phage Libraries

Alongside the installation of novel chemical moieties through genetic code expansion, several different bioconjugation reactions have been employed to diversify phage-displayed peptides. There have been numerous reactions developed for introducing new functional groups into proteins. While there have been a few studies that nonspecifically label phages with fluorophores or clickable handles (usually on pVIII), this section will be focused on selectively labeling phage-encoded libraries.^122−124^ The main difficulty in labeling phage libraries revolves around tuning the specificity of the compounds to modify the desired peptide without affecting pIII and other phage coat proteins that are required for proper infectivity. In addition to concerns involving selectivity of chemical modifications, it has also been challenging to directly quantify labels on phage. Especially when libraries are displayed on pIII, the individual proteins are typically at concentrations that are too low to be detected by mass spectrometry. Therefore, several different indirect methods are used to quantify phage modifications, such as ELISA, biotin pulldown, and activity assays. These indirect techniques make it difficult to identify exactly which peptides are being modified on phage and to what degree they have been modified. While these are ongoing issues, there have been several successful campaigns over the past 20 years that selectively modify phage libraries and identify therapeutic peptides via phage display.

**Figure 7:**
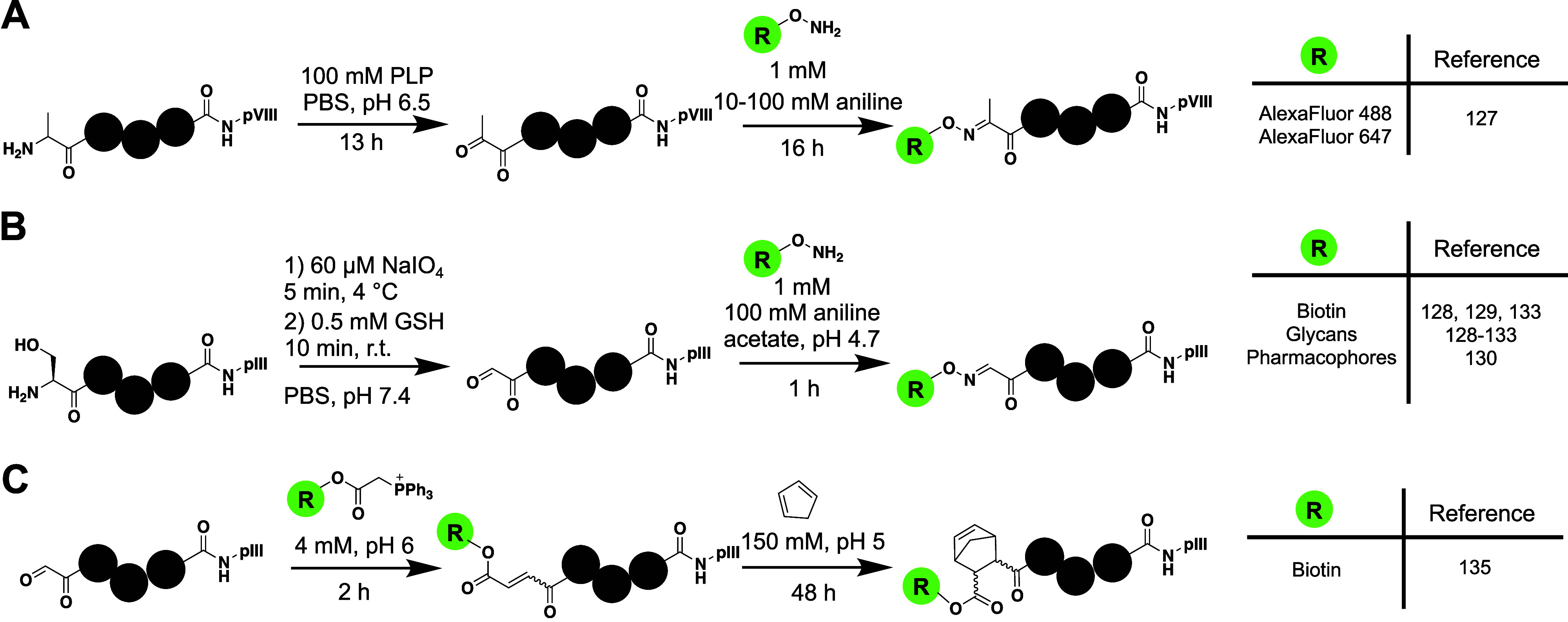
Functionalization of ketones and aldehydes on phage. A) Generation of ketones on phage by transamination allows for functionalization via an oxime ligation with alkoxyamines. B) The N-terminal serine can be converted into glyoxylate by oxidative cleavage with NaIO_4_ and the afforded aldehyde has high reactivity toward alkoxyamines. The resulting aldehydes have been functionalized with a wide variety of moieties. C) Functionalization of phage-displayed libraries containing N-terminal serines through Wittig and Diels–Alder reactions.

Most methods for selectively incorporating unnatural modifications to phage involve taking advantage of N-terminal residues. Because the phage coat proteins typically have N-terminal periplasmic tags that are cleaved during packaging, N-terminal amino acids other than methionine can be easily generated. One of the first techniques to install novel modalities into phage libraries involved native chemical ligation to introduce peptides onto the N-termini of pIII and pVIII.^125^ By genetically encoding an N-terminal cysteine to pVIII or pIII, they were able to use thiophenol for ligation of peptide chains containing the ncAAs kynurenine and norvaline to each of the phage proteins. Another strategy developed by Carrico et al. selectively modified N-terminal residues using pyridoxal-l-phosphate (PLP) to catalyze a transamination reaction and generate N-terminal ketones (Figure 7A).^126^ Following generation of a ketone, they were then able to successfully label phage proteins with alkoxyamine-fluorophores. MALDI-MS demonstrated efficient, selective labeling of the N-terminus of pVIII at pH 6.5 in spite of two lysines also being available for modification in pVIII. With the addition of 100 mM aniline, the reaction proceeded to label approximately 80% of pVIII with fluorophores. However, labeling was also observed on all other coat proteins with accessible N-termini (pIII, pVII, and pIX).^126^ To create productive phage libraries for biopanning, more efficient and selective labeling should be desired.

### 3.2 Oxime-based Ligation into Libraries Containing N-terminal Serines or Threonines

In a similar manner to the generation of N-terminal ketones and subsequent substitution of alkoxyamine derivatives on phage, there have been several studies that have taken advantage of N-terminal serine and threonine to selectively introduce new moieties onto pIII. The N-terminal serine or threonine can be converted into an *N*-glyoxylate that contains an aldehyde functional group through oxidative cleavage by sodium periodate.^127^ The Derda Lab pioneered application of this reaction to phage libraries and has thoroughly demonstrated its effectiveness in introducing a variety of unnatural modifications. Introduction of glycopeptide libraries onto phage-displayed peptides has been employed through generation of N-terminal aldehydes, followed by oxime ligation with aminooxy-derivatized glycosides (Figure 7B).^128^ Elegant biotin pulldown assays allowed for the kinetics of the reaction to be characterized thoroughly on phage. After 5 min of reaction with NaIO_4_, nearly all phages were converted to the reactive aldehyde intermediate (*k* = 210 M^–1^ s^–1^). Addition of aniline in the subsequent step (100 mM aniline acetate, pH 4.7) increased the oxime ligation nearly 100-fold, resulting in an observed rate on phage of 3.3 M^–1^ s^–1^.^128^ They demonstrated approximately 80% labeling only on phages containing the N-terminal serine and also showed effectiveness in labeling a commercially available heptapeptide library (Ph.D. 7).^128^ The resulting glycopeptides from this method resemble natural *N*- and *O*-linked glycans, giving them promise in identifying peptide ligands for lectins and other carbohydrate-binding proteins.^129^ The Derda Lab has taken advantage of this technique to guide phage-based selections toward active sites by modifying phage libraries with glycans,^130−133^ sulfonamides,^130^ and biotin.^130,134^ They have also combined these modifications with integration of silent barcodes downstream of the peptide library, allowing for the ability to simultaneously screen libraries containing different N-terminal modifications. In doing this, they have identified peptide ligands with low nanomolar potency for carbonic anhydrase and femtomolar peptides for streptavidin (Table S1, Peptides 34–48). This study also observed selective identification of ligands dependent upon the compound that the N-terminus is functionalized with.^130^ Libraries modified with glycans show good potential to identify peptides for lectins and other proteins with affinities for oligosaccharides. In particular, an arabinose-modified library was used to isolate peptides with low micromolar affinity for CS-35 Ab, an antibody that binds to an arabinose-containing mycobacterial antigen (Table S1, Peptides 49–52).^131^ Similar studies using mannose and galactose-modified N-termini have demonstrated the ability to isolate glycopeptides with micromolar affinity for DC-SIGN (an antigen recognition protein in dendritic cells) and phages with affinity for Galectin-3 (Table S1, Peptides 53–56).^132,133^ The Derda Lab has also used the afforded aldehyde from NaIO_4_ oxidation to investigate ligands for the Wittig reaction and install dienophiles onto phage libraries that can subsequently be modified through Diels–Alder reactions (Figure 7C).^135^ One consideration is that when using aniline as catalyst for the oxime ligation, the intermediate Schiff base undergoes quick hydrolysis, resulting in irreversible loss of the original aldehyde.^136^ To prevent this degradation on phage, Derda et al. developed benzamidoximes that react quickly (*k* = 40 M^–1^ s^–1^) with aldehydes without the addition of aniline.^136^ Modification of phage libraries through N-terminal aldehydes demonstrates the effectiveness of chemically functionalizing phage; however, the requirement of harsh oxidative conditions and multiple buffer exchanges may be undesired for some studies.

### 3.3 Post-translational Chemical Modification of Cysteine-Containing Phages

**Figure 8:**
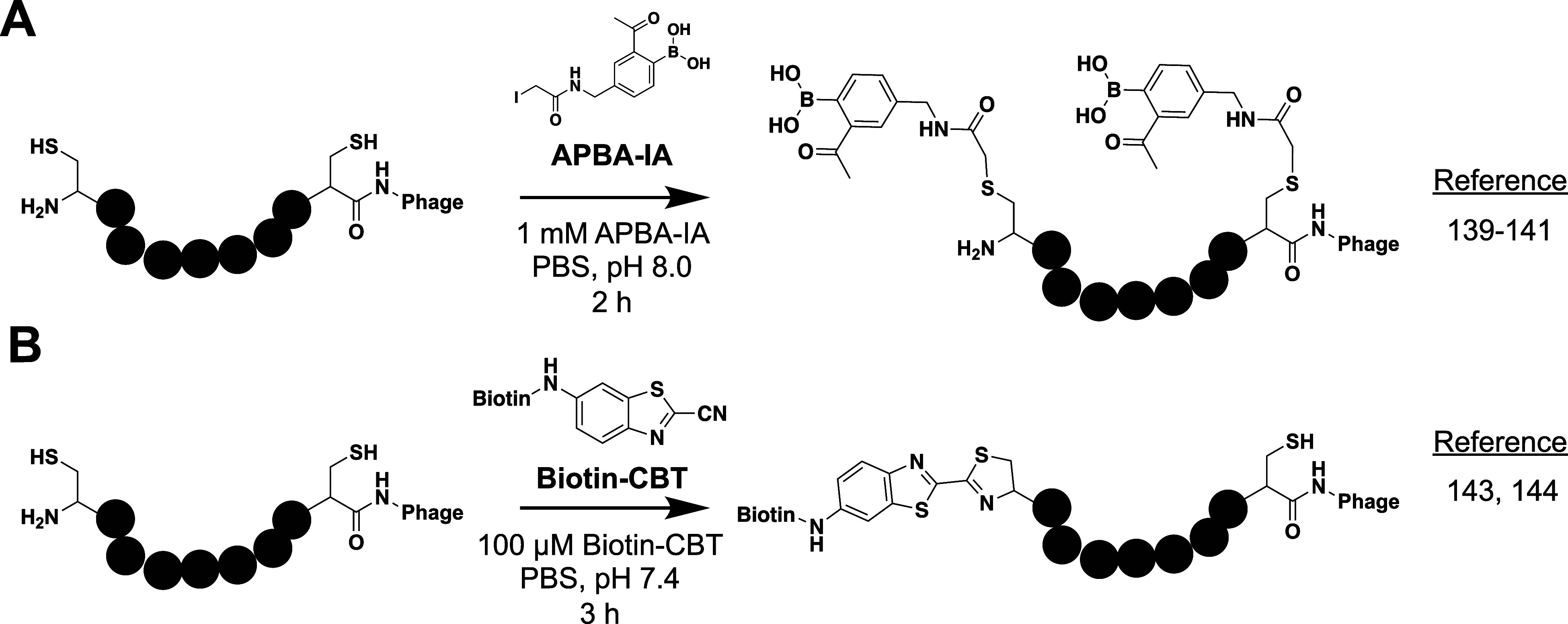
Modifications of cysteine residues on phage. A) Iodoacetamide derivatives react quickly, but nonspecifically with thiols on phage. These were used to install boronates for reversibly covalent functionalization of phage libraries. B) N-Terminal cysteines can be selectively modified by electron-deficient nitriles, such as Biotin-CBT, that quickly condense to thiazolidines at neutral conditions.

In addition to chemically modifying the N-terminal serine and threonine, there have been several studies that take advantage of cysteines to functionalize phage libraries. There are multiple biocompatible electrophilic reagents that have been developed to react nonspecifically with cysteines in proteins. With the abundance of cysteine being relatively low in proteins, it makes it a good candidate for phage modification even if a reaction is nonselective. However, there are key disulfide bonds in pIII that result in decreased infectivity when modified. Two different techniques have been established to mitigate this issue. One innovation that helped to reduce off-target concerns when modifying cysteines in phage libraries was the development of phages that lack disulfide bonds in pIII. Schmid et al. employed an *in vitro* evolution strategy to select for proteolytically stable pIII variants, ultimately identifying pIII proteins that lacked disulfide bonds.^137^ Although the disulfide-free phage showed lower infectivity, it was crucial in allowing for modifications of cysteine-containing peptide libraries. Additionally, the use of immobilized TCEP allows for selective reduction of cysteines within peptide libraries on phage, rather than the wild-type disulfides within pIII.^138^ Zheng et al. used the immobilized TCEP to selectively reduce cysteines on a CX_7_C library displayed on pIII, and then selectively reacted the two cysteines with (2-acetyl-4-((2-iodoacetamido)methyl)phenyl)boronic acid (APBA-IA, Figure 8A).^139^ Incorporation of APBA into the phage library allows for reversible covalent interactions with amines under biological conditions, and they took advantage of this to develop selective peptide antibiotics/probes for amine-presenting bacteria (Table S1, Peptides 57–67).^139−141^ This study highlights the potential applications of using cysteine-reactive reagents to modify phage, yet more selective reactions should be desired to avoid potentially modifying cysteines within the library or in other phage proteins.

To develop cysteine-modifying reagents with higher selectivity, most studies have looked to differentiate the N-terminal cysteine from an internal cysteine. This was inspired by previous development of cyanobenzothiazole (CBT) reagents that quickly condense (*k* = 9.19 M^–1^ s^–1^) with N-terminal cysteines at physiological conditions to generate thiazolidine adducts to proteins.^142^ Although primarily focused on generating macrocyclic peptide libraries on phage, two different studies have demonstrated biotin labeling of N-terminal cysteine-containing phages using biotin-CBT (Figure 8B).^143,144^ An additional study has also demonstrated similar reactivity using biotinylated 2-((alkylthio)(aryl)methylene)malononitrile (TAMM) derivatives that form thiazolidine adducts with the N-terminal cysteine.^145^ Biotin pulldown assays using these reagents indicated approximately 70% labeling of the peptide libraries.^143−145^ All of these labeling experiments were performed at neutral pHs in standard phage buffers. Although these only demonstrated biotinylation of peptide libraries, we envision that similar strategies could be used to further functionalize peptide libraries with diverse functional groups, such as glycosides, lipopolysaccharides, or pharmacophores. In comparison to the originally developed oxime ligation techniques, condensation on N-terminal cysteines provides a more straightforward labeling strategy that eliminates buffer exchanges and multiple phage precipitation steps. One strategy has combined the cyanobenzothiazole approach with alkylation of an internal cysteine to have selective double labeling of N-terminal and internal cysteines, respectively.^146^ The double labeling afforded integration of two covalent warheads to a CX_9_C library displayed on pIII. To do this, they first installed an APBA-CBT derivative by reacting it with an N-terminal cysteine. Then, they reacted the phage library with an iodoacetamide containing α-cyanoacrylamide to label the internal cysteine residue. This gives the ability to covalently interact with amines and cysteines on target proteins. Through biotin pulldown assays, they demonstrated at least 50% efficiency for labeling the N-terminal residues and nearly complete labeling of the internal cysteine.^146^ They then used the double-labeled library to select for peptides that bound to TEV protease in the low micromolar range (Table S1, Peptides 68–73). While these are promising avenues for creating new modalities in phage-displayed peptide libraries, most other cysteine-based reactions have been tailored for generating cyclic peptide libraries on phage and this will be discussed in detail in the following sections.

### 3.4 Post-translational Modifications for Generating Phage-Displayed Cyclic Peptide Libraries

Cyclic peptides show increased therapeutic potential in comparison to their linear counterparts due to several factors, such as having more rigid structures, having possibly enhanced membrane permeability, and being less susceptible proteolysis.^104,147−149^ Many efforts have attempted to use phage display for the identification of cyclic peptides that bind to therapeutic targets. Initial displayed cyclic peptides libraries were generated in situ through disulfide bond formation that forms spontaneously within the periplasmic space.^150,151^ However, the use of peptides containing disulfides is potentially problematic, as they are unstable in reducing environments. Because of this, several studies have investigated the development of reagents that create stable cyclic peptide libraries on phage. In addition to creating cyclic peptide libraries on phage, reagents used for cyclization can also be functionalized to display other moieties and install new reactivities to the phage libraries. The majority of reported techniques rely on nonspecifically reacting with cysteines displayed on phage; however, there have been a few recent strategies to develop cyclic peptide libraries using reagents that selectively modify N-terminal amino acids.

**Figure 9:**
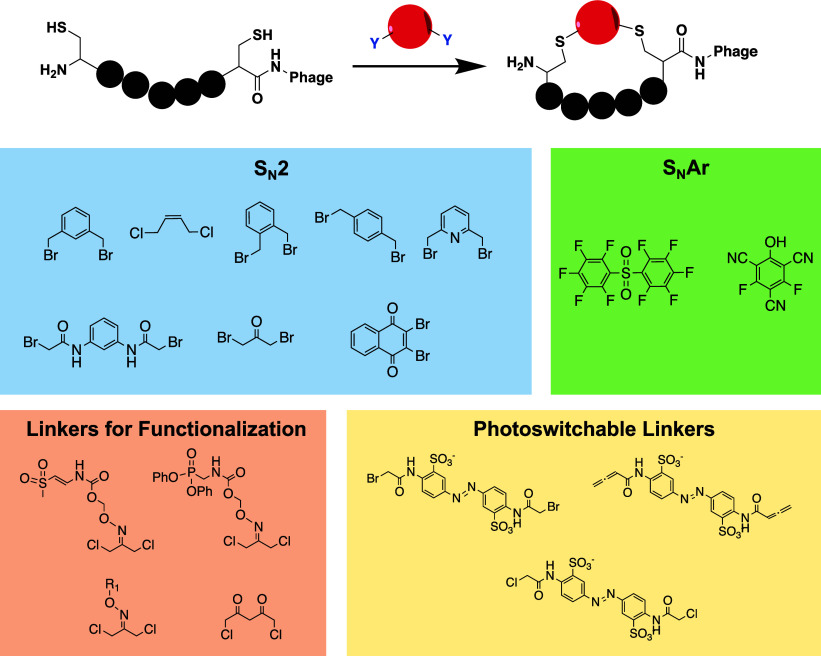
Peptide cyclization on phage by highly reactive bis-electrophiles. Phages have traditionally been cyclized via two cysteines that react with activated halides (Y = Br, Cl, I) through S_N_2 or S_N_Ar reactions, with the exception of one allene-based derivative. Representative reactive moieties are grouped by reaction type or additional functionalization of the peptides.

The most standard approach involves using alkylating reagents that are composed of highly reactive bis-electrophiles to react with two cysteines on phage libraries, resulting in cyclization of the displayed peptides (Figure 9). A diverse amount of electrophiles are available for labeling cysteine residues on phage. Although they typically react nonspecifically with cysteine residues, the use of phages that contain disulfide-free pIII proteins can allow for modification of the library region without reductions in infectivity.^137^ Due to the promising abilities of α-helical peptides, several groups developed linkers to chemically synthesize stapled peptides using cysteine alkylating reagents in the early 2010s.^152−154^ Similarly, the first reported molecules for cyclization of peptides on phage consisted of alkyl and benzyl halides that looked to stabilize alpha-helices on phage libraries.^155^ Heinis et al. attempted to stabilize α-helices by modifying phages at *i* and *i*+4 cysteine residues with α,α′-dibromo-*m*-xylene (mDBMB, Table 3), *trans*-1,4-dibromo-2-butene (TDBB), and *cis*-1,4-dichloro-2-butene (CDCB, Table 3).^155^ Through circular dichroism studies of a model peptide, they observed efficient formation of α-helices only with those reacted with mDBMB and CDCB. Phage selections using both CDCB and mDBMB then afforded low nanomolar ligands for β-catenin (Table S1, Peptides 74–78). Shortly after this initial study, the Heinis Lab developed several alkyl bromides to create cyclic peptide scaffolds on phage (oDBMB, pDBMB, mDBMP, DBAmB, DBAc, and DBPyD; Table 3). Interestingly, they took advantage of reacting the bis-electrophiles with libraries containing four cysteines to form double-bridged peptides.^156^ Through comparative phage selections against plasma kallikrein, they observed preferential enrichment of libraries dependent upon the organic linker used in selection, indicating modification of the phage libraries. In doing this, they identified low nanomolar ligands for IL-17 (Table S1, Peptides 79 and 80), an interleukin target that binds to some currently approved antibody drugs.^156,157^ The same library was also used to identify subnanomolar inhibitors of plasma kallikrein (Table S1, Peptides 81 and 82) with enhanced specificity for the protein.^156^ These same reagents have also resulted in the identification of potent cyclic peptide inhibitors (Table S1, Peptides 83–93) for other kallikrein-related peptidases (KLK5 and KLK7) using the same four-cysteine library approach.^158^

**Table 3:**
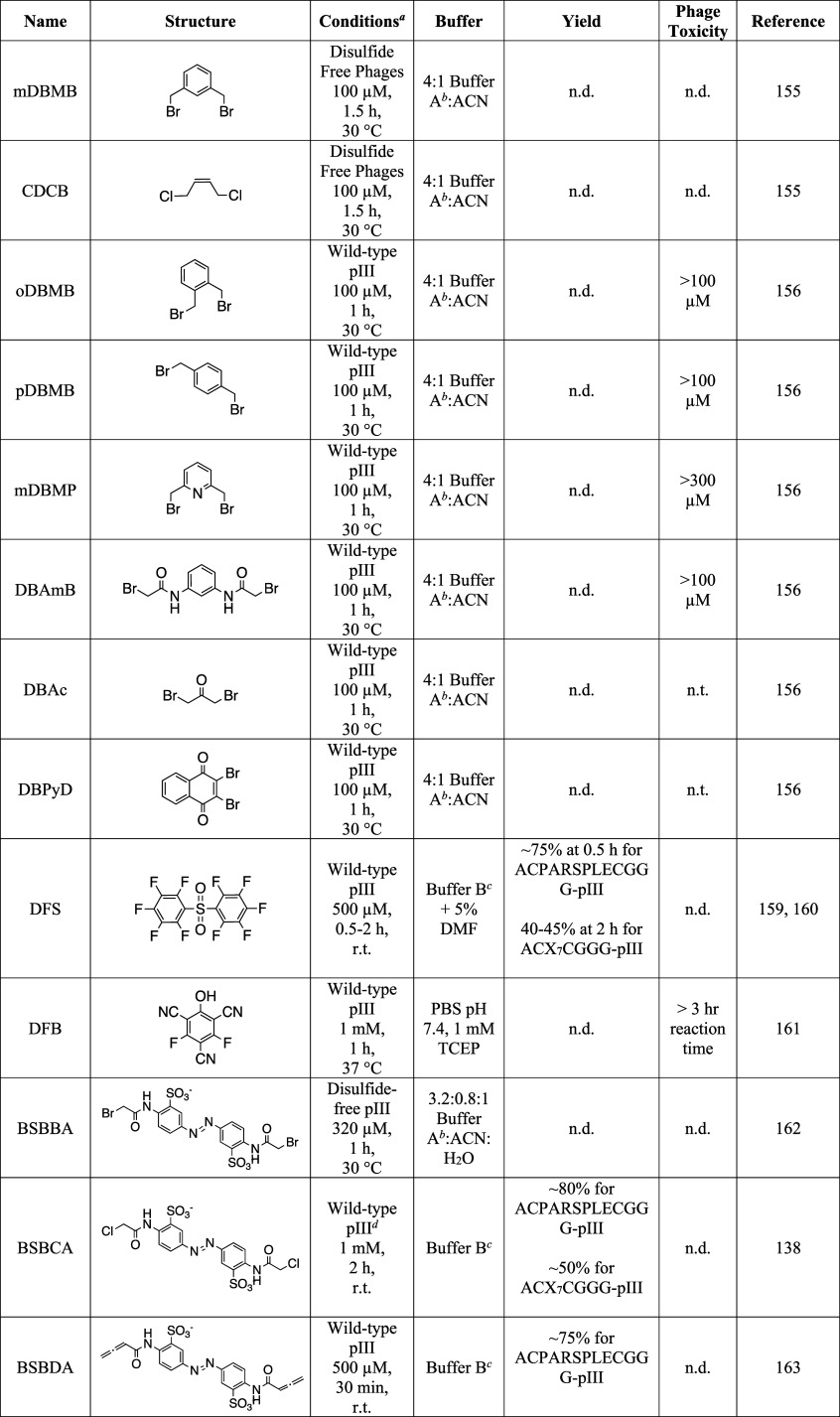

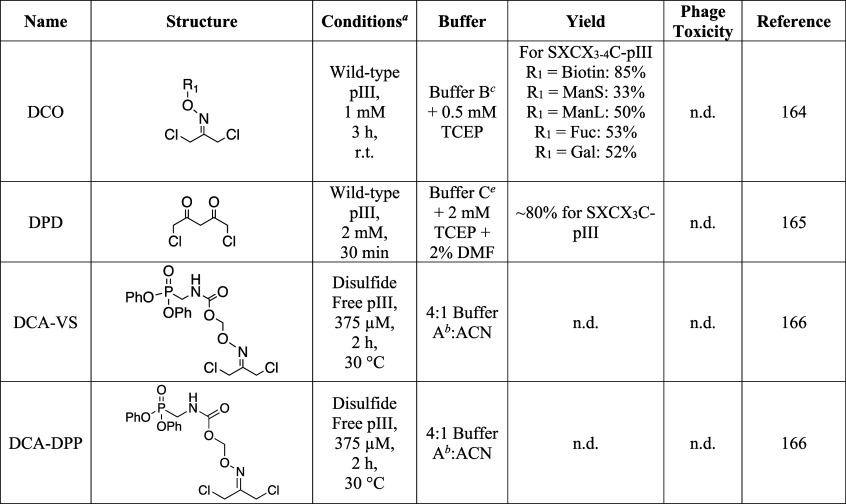
Symmetrical Bis-Electrophiles for Cyclization of Two-Cysteine Phage Libraries

a All phages were reduced using TCEP prior to cyclization. See reference for specific conditions.

b Buffer A: 20 mM NH_4_CO_3_, 5 mM EDTA, pH 8.

c Buffer B: 50 mM Tris pH 8.5.

d Phages reduced with iTCEP for 48 h.

e Buffer C: 55 μL of PBS (phage), 36 μL of water, 10 μL of 1 M sodium bicarbonate, pH 8.5.

In addition to traditional S_N_2 reactions with cysteines on phage, S_N_Ar substitutions have also been successfully employed to cyclize phage displayed libraries with two cysteines (Figure 9). The first of these applications took advantage of a decafluoro-diphenylsulfone (DFS, Table 3) to react with cysteines at extremely quick kinetics with rates upward of 180 M^–1^ s^–1^.^159^ A promising advantage of developing peptide ligands using these perfluoro compounds is their ability to be directly detected for binding and *in vivo* analysis through ^19^F NMR.^160^ Another similar strategy that takes advantage of S_N_Ar reactivity involves using 2,4-difluoro-6-hydroxy-1,3,5-benzenetricarbonitrile (DFB, Table 3) to cyclize phage libraries with two cysteines.^161^ Although it is slower than the reaction with DFS, the kinetics are sufficient for effective phage labeling with a second order rate constant of 9.2 M^–1^ s^–1^. Both linkers have proven useful in the phage-assisted identification of cyclic peptides, with the resulting peptides (Table S1, Peptides 94 and 95) binding with micromolar affinity to human serum albumin and Bcl-xl.^160,161^

In addition to simply creating macrocyclic peptides on phage, cyclization of peptides with organic linkers also allows for further functionalization of peptide libraries. By incorporation of additional moieties into the linkers, additional properties can be given to the peptide libraries that facilitate selections for certain proteins. In two separate studies, alkylation of cysteines using chloroacetamides (BSBCA, Table 3)^138^ and bromoacetamides (BSBBA, Table 3)^162^ has been used to generate light-responsive genetically encoded cyclic peptide libraries by incorporating an azobenzene core within the organic linker. These allow for the potential to create photoswitching peptides that can activate or deactivate in the presence of UV light, which have proven useful in developing probes for a variety of assays. The BSBCA linkers showed only 50% reactivity on the phage and did not produce high affinity peptides in selections with affinities in the range of 300–500 μM for streptavidin (Table S1, Peptides 96–98). However, the BSBBA functionalized library resulted in peptides with low micromolar affinity for streptavidin (Table S1, Peptides 99–102).^162^ Interestingly, Heinis et al. performed their selection to identify peptides that were activated by UV light, rather than inactivated, which may have contributed to more successful binding and inhibition.^162^ Photoswitchable libraries have also been made through reactivity with a bis(allenamide) functionalized azobenzene (BSBDA, Table 3). This derivative reacts significantly quicker than the previously established alkyl halide reagents with rates on the order of 16–30 M^–1^ s^–1^ and reactions with reduced phages are near completion after only 20 min of incubation.^163^ Because of its increased reactivity, application of BSBDA to phage selections may result in more efficient production and selection of phage-displayed photoswitchable libraries.

There have been multiple studies that have created unnatural motifs on phage-displayed libraries by using bis-electrophiles that react with cysteine. For example, Derda and co-workers have used dichloro-oxime (DCO, Table 3) derivatives to generate glycan-modified macrocyclic peptide libraries on phage.^164^ A diketone-containing organic linker has also been used to generate cyclic peptide libraries containing pharmacophores to direct phage selections. To do this, a dichloropentadione (DPD, Table 3) derivative first reacted with two cysteines on the phage to afford cyclic peptide libraries.^165^ Biotin pulldown assays indicated approximately 75% efficiency for introduction of the diketone to the phage libraries. Following cyclization, the linker was then functionalized with pharmacophores containing hydrazines that can condense with the diketone, ultimately forming cyclic peptides bearing pharmacophores through a diazole linkage.^165^ Hydrazine reactivity with the diketone proceeds quickly at pH 5.0, with the reaction completing after only 1 h. The pharmacophore-functionalized libraries successfully identified ligands with low nanomolar affinity for carbonic anhydrase (Table S1, Peptides 103 and 104). While this is an excellent display of functionalizing cyclic peptide libraries through macrocyclic linkers, it is hindered by the generation of regioisomers during the hydrazine condensation with diketones. Similar reactions that are symmetric in nature would simplify characterization and identification of hit peptides following selections.

Another interesting design has been the introduction of covalent warheads into phage-displayed cyclic peptide libraries through derivatized macrocyclic linkers. Bogyo et al. introduced vinyl sulfone and diphenylphosphonate into cyclic peptide libraries that were cyclized with a derivatized dichloroacetone linker (DCA-VS and DCA-DPP, respectively; Table 3).^166^ The afforded DCA-VS and DCA-DPP functionalized libraries were then successfully used to identify inhibitors of TEV protease and FphF, respectively, with IC_50_ values in the low micromolar range (Table S1, Peptides 105 and 106). In a similar approach by Gao et al., cyclic peptide phage libraries were modified by a dichloro-oxime derivative to contain 2-acetylphenyboronic acids (APBAs) for covalent targeting of lysine residues. The APBA-functionalized library was used to identify peptides that inhibited sortase A at micromolar potency and for identifying peptides to detect the SARS-CoV-2 spike protein (Table S1, Peptides 107–109).^167^

**Figure 10:**
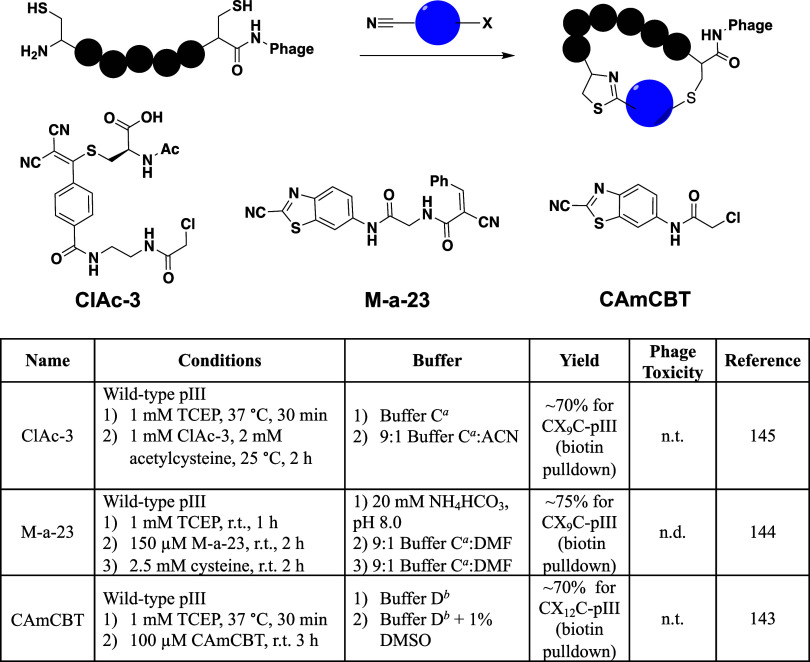
N-Terminal cysteine directed cyclization of phage displayed peptide libraries. Three reagents have been developed that take advantage of condensation with N-terminal cysteines to provide selectivity for cyclization of peptide libraries. ^*a*^Buffer C: 50 mM PBS, pH 7.4. ^*b*^Buffer D: 10 mM HEPES, 150 mM NaCl, 10 mM MgCl_2_, 1 mM KCl, pH 7.4.

Although there have been a broad range of modifications employed to generate cyclic peptide libraries through highly reactive bis-electrophiles, they are potentially limited in a few capacities. First, all of the above-mentioned bis-electrophiles are agnostic in their selectivity for cysteines, resulting in them necessarily being symmetrical. This limits functionalization and hinders further diversification of phage libraries. Additionally, the lack of specificity for labeling may result in less efficient display of the desired cyclic peptide libraries, with different modifications occurring elsewhere on the phage proteins. To counter these issues, a few recent studies have looked to take advantage of N-terminal residues for generating cyclic peptide libraries more selectively and allow for asymmetrical designs. The first example of potentially asymmetric labeling involved taking advantage of N-terminal cysteines to selectively modify phage libraries. Wu et al. designed a cyclic peptide linker (ClAc-3, Figure 10) that introduced a chloroacetamide moiety into 2-((alkylthio)(aryl)methylene)malononitrile (TAMM) derivatives.^145^ In doing this, the TAMM condensation reaction with the N-terminal cysteine directed reactivity to the displayed N-terminal cysteine-containing library. Following this reaction, a proximity-driven cyclization event occurs between an internal cysteine and the chloroacetamide. In this method nonspecific labeling should be minimal, as the rate for chloroacetamide labeling is low (>0.05 M^–1^ s^–1^) at neutral pH and no other phage proteins contain N-terminal cysteines. Through biotin pulldown assays, they demonstrated approximately 75% reactivity on phage and observed no significant phage toxicity. Biopanning of a CX_9_C cyclic peptide library to Bcl-2 resulted in the identification of cyclic peptides with mid nanomolar affinity for the protein (Table S1, Peptides 110–112). The study also identified peptides for MDM2 and Keap1 with nanomolar potency (Table S1, Peptides 113–116). Two similar strategies have also been developed that take advantage of the cyanobenzothiazole (CBT) condensation reaction with N-terminal cysteines to selectively cyclize phage libraries.^143,144^ These also resulted in similar efficiencies (60–75%) for modifying N-terminal cysteine containing libraries with the CBT derivatives. The main difference in these two involved the internal cysteine reactive moiety—Liu et al. reported using a chloroacetamide-containing CBT (CAmCBT, Figure 10) that reacts irreversibly,^143^ while Gao et al. functionalized CBT with a reversibly reacting α-cyanoacrylamide (M-a-23, Figure 10).^144^ Because of its reversible nature, peptides cyclized with the α-cyanoacrylamide did exhibit some free cysteine after cyclization, albeit the reverse reaction occurred at a low rate. To counter this, they demonstrated that the peptides could be synthesized without the nitrile to prevent the reversible reaction, although the nitrile is necessary for phage reactivity.^144^ M-a-23 was successful in producing macrocyclic peptide ligands for the Keap1 Kelch Domain, Sortase A, and streptavidin (Table S1, Peptides 118–121).^144^ CAmCBT also proved useful in creating a neutralizing macrocyclic peptide with low micromolar affinity for the Spike protein that prevented SARS-CoV-2 infection (Table S1, Peptide 117).^143^ An additional strategy has also been reported that takes advantage of an internal cysteine and reductive amination of the N-terminus to generate asymmetric linkers for macrocyclic peptide libraries.^168^ In this study, Mayer et al. developed two different aldehyde derivatized benzyl bromoacetamides that displayed asymmetric cyclization of libraries consisting of AX_8_C peptides fused to pIII. The reaction was performed in two steps: 1) alkylation of the cysteine with bromoacetamide 2) followed by reductive amination with NaBH_3_CN. The library was screened against streptavidin and proved to efficiently identify a peptide with low nanomolar affinity (Table S1, Peptide 122). In comparison to the approach taking advantage of N-terminal cysteines to direct cyclization, this strategy is likely less selective, as lysines within the library could also react via reductive amination. Nonetheless, all of these studies open up new avenues in selective peptide cyclization on phage and allow for asymmetric functionalization of macrocyclic libraries.

### 3.5 Post-translational Modifications to Generate Bicyclic Peptides

**Figure 11:**
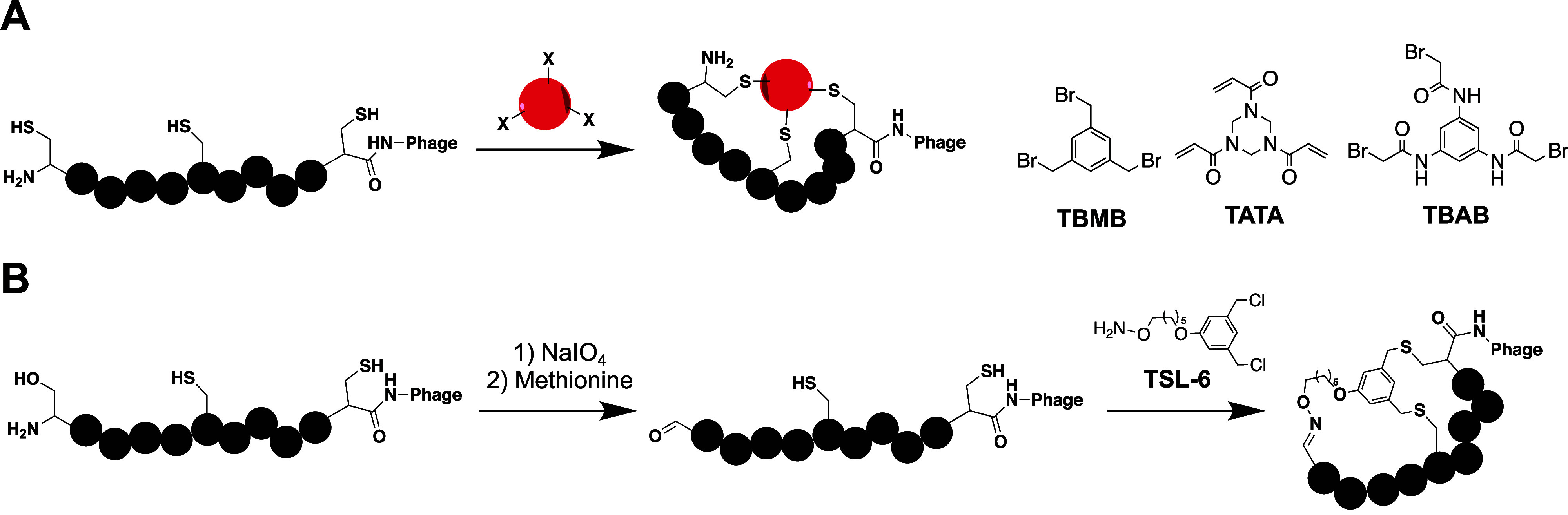
Generation of phage-displayed bicyclic peptides. A) Trisymmetric linkers containing cysteine-reactive electrophiles were initially developed to create bicyclic peptide scaffolds on phage. B) N-terminal serine selective bicyclic linkers have been recently developed to screen bicyclic peptides through a two-step reaction following oxidation of the N-terminal serine with sodium periodate.

Just as the constraints in monocyclic peptides result in improved pharmacokinetics, the generation of bicyclic peptides as either a fused bicycle or double-bridged peptide adds conformational constraints for even more rigidity in comparison to monocyclic peptides. This subsequently results in improved binding affinities and resistance to proteolysis. In this section, we will review methods for producing bicyclic peptide libraries on phage and look into applications that demonstrate these enhanced properties. The Heinis Lab has developed and characterized several different molecules that modify cysteines on phage to produce bicyclic peptides (Figure 11A). All of these are symmetrical electrophiles that contain activated halides for reaction with cysteines through S_N_2 or Michael Addition reactions. Initially, a trisymmetric derivative, TBMB (Table 4), was used to create bicyclic peptide libraries by reacting with three cysteines displayed on the phage.^169−172^ While TBMB demonstrated complete reactivity with a peptide–protein fusion after only 1 h reaction at 30 °C, there was a considerable loss in phage infectivity when incubated with more than 10 μM compound. Considering the highly electrophilic nature of benzyl bromides, this is likely due to reactivity with other nucleophilic residues on the phage coat proteins. Despite this toxicity, low nanomolar peptide inhibitors for kallikrein were developed using TBMB and they demonstrated high selectivity for the bicyclic form (Table S1, Peptide 123).^169^ Following these initial studies, TBMB has proven to be a linker for a variety of bicyclic peptide sizes, ranging from 3 to 6 amino acids between each cysteine, and the cyclization appears to enhance binding activities in comparison to disulfide-cyclized peptides.^170,173^ One study used TBMB to identify peptides (Table S1, Peptides 124 and 125) with micromolar affinity for uPA, a serine protease upregulated in many tumors.^170^ In addition to peptides fused to phage, TBMB has also proven to be effective at displaying bicycles on antibody Fc domains, although some side reactivity was observed that resulted in dimerization of the antibody fragments.^174^ While TBMB is an effective linker for producing bicyclic peptides on proteins, it appears to be less effective when reacting with phages, only generating the desired bicyclic peptide on 10% of phages and showing considerable toxicity to wild-type phages above 10 μM compound.^171^ Despite this apparently low reactivity, it has still been successfully used in numerous selections to identify bicyclic peptide ligands for proteins.^169,175−179^ It may be worth exploring additional ways to verify the reactivity on phage, such as with biotin pulldown assays.

**Table 4:**
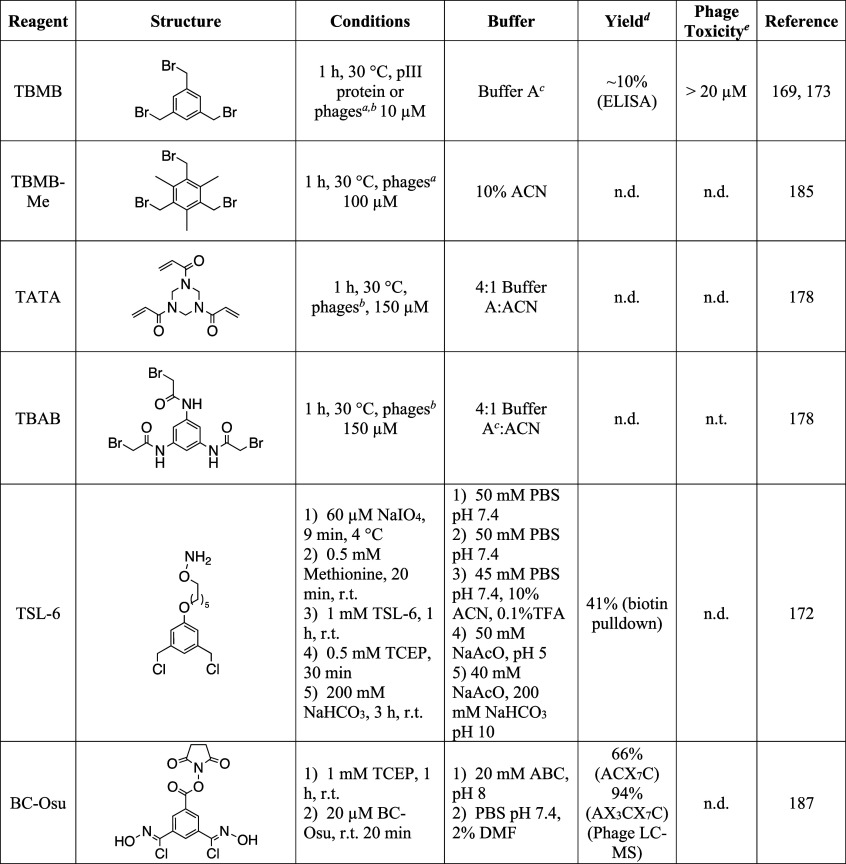
Reagents to Generate Bicyclic Peptide Scaffolds on Phage

a Wild-type phage.

b Disulfide-free phage mutant.

c Buffer A: 20 mM ABC, 5 mM EDTA, pH 8.

d n.d.: not determined.

e n.t.: No observed toxicity.

There have been several campaigns to improve upon the initially developed bicyclic linker TBMB both by modifying the central core and the electrophilic moieties. Bicyclic peptides exhibit different conformations depending upon the central linker used, so the development of additional linkers is expected give access to novel structures of peptides.^180^ The first variations looked to generate more hydrophilic cores to promote interactions between the peptide libraries and the target proteins, with the core pointing toward the solvent.^178^ They developed two hydrophilic linkers, 1,3,5-triacryloyl-1,3,5-triazinane (TATA, Table 4) and *N*,*N′*,*N′*-(benzene-1,3,5-triyl)-tris(2-bromoacetamide) (TBAB, Table 4), that contained hydrophilic cores fused to cysteine reactive groups. As expected, the acrylamide moieties in TATA showed considerably slower reactivity than TBMB or TBAB, with reactions needing 40 μM compound to completely cyclize a model pIII fusion protein.^178^ Additionally, when using acrylamide derivatives to react with cysteine, it is necessary to remove all of the TCEP in solution before cyclization to prevent a Phospha-Michael addition of TCEP.^181^ Despite having a similar mode of action to TBMB, there was no observed phage toxicity when incubating disulfide-free phages with 160 μM TATA or TBAB.^178^ Bicyclic peptides isolated from phage selections using the three molecules (TBMB, TATA, and TBAB) exhibited enhanced activity (up to 1000-fold higher) when cyclized with the linker used during the selection, indicating a structural role for the linkers to play in peptide folding and protein interactions.^178^ To account for this, structural studies demonstrated efficient hydrogen bonding between the linkers and the peptide backbone and/or amino acid residues.^178,180^ These additional interactions can potentially stabilize peptides in the bicyclic form and promote affinity to desired targets. With this increased potential and lowered phage toxicity, TATA and TBAB appear to be superior organic linkers for generating bicyclic peptides on phage. Nevertheless, there are some limitations to these two compounds, such as the necessity to have symmetrical linker cores and the nonspecific reactivity of the electrophilic regions. In addition to using a single bridge compound to generate bicyclic peptides from phage libraries containing three cysteines, Heinis et al. have also generated bicyclic peptide libraries using peptides containing four cysteines in combination with either two bis-electrophiles or disulfide bonds.^170,171^ In one interesting application, generation of a double-bridged peptide library using DBAc (Table 3) afforded identification of proteolytically stable peptides (Table S1, Peptides 126 and 127) with low nanomolar affinities for FXIa and IL-23R.^171^ This could be an exciting avenue to directly screen peptides that show good stability *in vivo*.

There are numerous applications of these bicyclic peptide linkers in drug discovery campaigns for a wide variety of targets, demonstrating the particular usefulness of these libraries. Peptides bridged using TBMB, TATA, and TBAB have been isolated in panning against β-catenin and have micromolar affinities (Table S1, Peptides 128–131).^175^ For the potential treatment of thrombotic diseases, a peptide cyclized using TBMB has been identified that binds the FXIIa with micromolar affinity (Table S1, Peptide 132).^176^ TBMB has also been used to identify possible antibiotic peptides through screening bicyclic peptides to bind Sortase A from *S. aureus*, with the optimized peptides giving low micromolar affinity (Table S1, Peptides 133–135).^177^ Studies have investigated cancer targets and identified bicyclic peptides that bind to oncoproteins serine protease uPA, Her2, Nectin-4, type-1 metalloproteases (MT1-MMP), and EphA2 using TBMB, TBAB, and TATA as linkers, with all of the optimized peptides showing nanomolar affinity for their respective targets (Table S1, Peptides 136–143).^178,179,182−184^ Bicyclic peptides have also been identified using a methylated TBMB (TBMB-Me, Table 4) to bind with high affinity to proteins associated with inflammatory disorders, such as TNFα (Tables S1, Peptide 144).^185^ These demonstrate the robustness of these bicyclic peptide libraries and their great potential to impact drug discovery efforts. Currently, the peptides identified for Nectin-4, MT1-MMP, and EphA2 are under clinical trials with Bicycle Therapeutics.

There have been multiple strategies to further optimize linkers for displaying bicyclic peptide scaffolds on phage. To avoid toxicities associated with the TBMB linker, a study was performed to optimize the TCEP reduction and coupling conditions that allowed for incubation using 100 μM TBMB, although there was still considerable toxicity of the linker (100-fold less infectivity). Although TBMB is the only compound that exhibited toxicity when incubated with phages, all three linkers blindly react with cysteines through highly activated electrophilic groups. This necessitates that they have trifold symmetry to avoid complications in peptide synthesis, which limits the potential diversity and modifications that can be introduced to the bicyclic peptides. Some strategies to work around these issues involve taking advantage of reactions that differentiate N-terminal amino acids from their internal counterparts. The first examples of this came from the Derda Lab, where an N-terminal serine was converted into glyoxylate by sodium periodate oxidative cleavage and the resulting aldehyde was reacted with an hydroxyl amine containing a dichloromethyl-benzene (TSL-6, Figure 11B, Table 4) for bicyclization with two internal cysteines.^172,186^ It may also allow for higher specificity in modifying phage cysteines within the library because less-reactive electrophiles can be catalyzed to react through proximity-driven interactions following the oxime ligation. Because of this, rather than being constricted to using disulfide-free pIII, wild-type pIII is compatible with this linker and higher phage yields can be achieved. Using this technique, the reported display of bicyclic peptides was 41% using a biotin pulldown assay, which is considerably higher than the reported 10% (ELISA assay) with TBMB.^171,172^ Conditions may still need to be optimized to achieve higher efficiencies of bicyclization. In addition to the improved selectivity of the linker, the displayed peptides also exhibit higher serum stability because they lack an N-terminus that can be recognized by proteases, and a recent report demonstrated the ability to generate proteolytically stable peptides for NODAL that inhibited in the micromolar range (Table S1, Peptide 145).^172^ A recently reported technique for generating bicyclic peptide scaffolds on phage involves using a compound (BC-Osu) with two chlorooximes and one NHS-ester to react with two cysteines and the N-terminus, respectively.^187^ The reaction exhibited high efficiency, with the desired bicyclic product forming with 66% and 94% yield in phage pIII proteins modified with ACX_7_C and AX_3_CX_7_C peptides on the N-terminus, respectively.^187^ While these yields are the highest reported for display of bicyclic peptides, BC-Osu is also limited by reacting nonspecifically. Because of this, it would be expected to modify not only the N-terminus of the library region on pIII, but also N-termini, lysines, and cysteines in other phage coat proteins. Thus, there is still a need to develop efficient, highly selective bicyclic linkers to afford the most useful phage-displayed bicyclic peptide libraries.

### 3.6 Outlook of Chemically Modified Phage Libraries

As discussed above, there have been many different techniques developed to chemically modify phage displayed peptide libraries. These have primarily been employed to generate novel moieties on phage for directing selections and to produce cyclic peptide scaffolds on the phage. The main challenge with modifying phage displayed libraries revolves around generating reagents that selectively modify the libraries rather than other phage coat proteins. We have highlighted several promising strategies on this front that look to selectively modify N-terminal cysteines and serines to direct the modifications to displayed peptide libraries. While these are efficient techniques, they usually show less than 75% labeling on the displayed peptides. However, this is expected since the labeling reactions are significantly affected by the chemical contexts surrounding the N-terminal cysteine and serine on both kinetics and thermodynamics. Given the extremely large library size, some of the N-terminal cysteines and serines might also be buried in the peptide contexts and therefore not accessible for labeling. Recently, site specific modification of the N-terminal glycine has been reported, which could allow for more quantitative production of pIII with an N-terminal glycine.^188^ As new bioconjugation approaches develop, they should be considered for use in phage displayed libraries.

Although there have been many approaches developed to modify phage libraries, there is still a need to develop standardized methods to directly characterize the modified products on phage. Usually, concentrations of pIII in phage libraries are in the femtomolar to nanomolar range, making them too dilute for normal mass spectrometry techniques. Because of this, indirect methods are used to quantify modifications on phage, such as ELISA and biotin pulldown assays. While these are innovative techniques that provide good insights into the modifications present, they still leave some ambiguity about the quality of the libraries that are displayed and actual modifications present. This ambiguity makes comparisons of reagents difficult and complicates quality control during biopanning experiments. Thus, novel mass spectrometry techniques allowing quantitative analysis of phage-displayed peptides would help immensely advance new labeling and cyclization methods for phage-displayed libraries.

## 4 Concluding Remarks for Phage-Display of Noncanonical Motifs

The identification of therapeutic peptides from unmodified phage-displayed libraries is hindered because linear peptides have poor pharmacokinetic properties. The introduction of ncAAs and motifs allows for a more simplistic transition of peptide lead compounds into therapeutics, as they can increase the stability and improve binding toward a desired target. In the past two decades, noncanonical peptides have been produced on phage-displayed libraries through two segregated techniques: genetic code expansion and chemical transformations. Both techniques have afforded highly diverse phage-displayed libraries, ranging from linear libraries containing noncanonical moieties for enhanced phage selections to display of cyclic peptide libraries for enhanced pharmacokinetics. To further diversify phage libraries, it may be worthwhile to combine the two fields. For example, introduction of ncAAs through genetic code expansion can give highly specific reactive handles to selectively label phage libraries and to produce specific cyclic peptide motifs that avoid the ambiguity of labeling natural residues with chemical reagents. This would afford highly diverse libraries that are selectively modified, increasing efficiencies of selections and peptide characterization.

While integration of genetic code expansion with chemically modified phage libraries would result in diverse scaffolds for phage-displayed peptides, there are still limitations to consider with phage display. The first of these involves the limitation regarding initial transformation efficiency—plasmids must be transformed into cells, which usually limits the displayed libraries to 10^10^ peptides. There has been a recent case that generated a library of 10^11^ peptides by transformation of diverse peptide scaffolds, but this would be the upper limit for most laboratories.^189^ One way that may get around this limitation would be the integration of phage-displayed peptides into *in vivo* evolution systems, such as phage-assisted continuous evolution, that simultaneously mutate and select for peptides with desired activities.^190^ This would provide essentially unlimited peptides available for screening, as higher diversities are generated as the evolution continues. Additionally, genetic code expansion in phage display is limited to only orthogonal translation systems, as the endogenous translation is necessary for producing all the phage proteins. Because of this, incorporation of ncAAs at sense codons is difficult due to competition with the natural amino acids. Adaption of phage display systems to *E. coli* with synthetic genomes may help to work around this limitation and further diversify the number of amino acids that can be incorporated. Nonetheless, phage display still is one of the most accessible techniques to develop peptide libraries, and the diversification of phage libraries gives opportunities to identify peptide ligands for various therapeutic targets.

Of the peptide display techniques, mRNA display has also demonstrated similar levels of success in identifying therapeutic peptides over the past 20 years. Although mRNA display can give libraries of up to 10^15^ peptides along with incorporation of highly diverse amino acids through use of flexizymes, there are several advantages in phage display that one should consider when deciding on a peptide display method. First, phages exhibit high stability in a wide variety of conditions (pH, temperature, proteolytic, etc.), which gives researchers the ability to diversify screening conditions.^26^ This also allows for the ability to perform selections on whole cells, organoids, tumors, tissues, and *in vivo*.^191−194^ In addition to enhanced stability, techniques that take advantage of the phage biology, such as the ability to enrich amber codons throughout the selection, can be very useful tools to enhance selections with ncAAs.^94^ Phage display also may be more accessible to certain research laboratories, as it requires simple molecular biology techniques in comparison to intricate *in vitro* translation systems. Display of larger proteins, such as nanobodies, may also be more practical using phage display, as the presence of puromycin in mRNA display could cause truncated protein expression.^195^ As development of peptide therapeutics has increased in the past 20 years, concurrent innovations in both mRNA display and phage display provide researchers with useful tools for *de novo* peptide drug discovery. It will be exciting to see future developments and applications as both techniques continue to address their current limitations.

## Supporting Information Available

Supplementary Table S1 includes the sequences and potencies of top selected peptides in works related to this review (PDF)
