# Supplementary material for: Diversification of Phage-Displayed Peptide Libraries with Noncanonical Amino Acid Mutagenesis and Chemical Modification
Source: Chem Rev. 2024 Apr 30;124(9):6051–77. doi: 10.1021/acs.chemrev.4c00004 (PMC11082904; doi:10.1021/acs.chemrev.4c00004)
Supplement: Supplementary file 1 — cr4c00004_si_001.pdf [file cr4c00004_si_001.pdf]

Supporting Information

Diversification of Phage-Displayed Peptide Libraries with Noncanonical Amino Acid Mutagenesis and Chemical Modification

J. Trae Hampton[a,*] and Wenshe Ray Liu[a,b,c,d,e,*]

[a]Texas A&M Drug Discovery Center and Department of Chemistry, College of Arts and Sciences, Texas A&M University, College Station, TX 77843, USA

[b]Institute of Biosciences and Technology and Department of Translational Medical Sciences, College of Medicine, Texas A&M University, Houston, TX 77030, USA

[c]Department of Biochemistry and Biophysics, College of Agriculture and Life Sciences, Texas A&M University, College Station, TX 77843, USA

[d]Department of Cell Biology and Genetics, College of Medicine, Texas A&M University, College Station, TX 77843, USA

[e]Department of Pharmaceutical Sciences, Irma Lerma Rangel College of Pharmacy, Texas A&M University, College Station, TX 77843, USA

*Correspondence should be addressed to J. Trae Hampton: jhampton1@tamu.edu and Wenshe Ray Liu: wsliu2007@tamu.edu

Table S1. **Selected Peptide Sequences Identified from Phage Selections Using Noncanonical Motifs**

Number	Peptide Sequence	Modifications	Target	Potency	Reference
1	ACHXHIMIC	Disulfide bridge<br>X = BpyA	Ni-NTA	1.4 µM	92
2	ACHHFXEHC	Disulfide bridge<br>X = BpyA	Ni-NTA	0.34 µM	93
3	VYAXYIF	X = mCF3F	TEV Protease	n.d.	94
4	VYFFXDE	X = mBrF	TEV Protease	n.d	94
5	KLXKHYP	X = mBrF	Streptavidin	n.d.	94
6	KMXPQRN	X = mBrF	Streptavidin	n.d.	94
7	CTVXTSL	X = BuK	SIRT2	68 nM	94
8	TCTVXIG	X = BuK	SIRT2	92 nM	94
9	YXFFWED	X = Aoda	HDAC8	5.3 µM	100
10	YDVYCYX	X = BuK	ENL	36 nM	101
11	WWIIEXG	X = BuK	ENL	881 nM	101
12	CHMXEYDNVLWEVC	Disulfide bridge<br>X = AllocK	ZNRF3	5.9 µM	103
13	CEVTXDYTWSVWPC	Disulfide bridge<br>X = BocK	ZNRF3	4.5 µM	103
14	CYSTSXHGYMWVTC	Disulfide bridge<br>X = BocK	ZNRF3	1.6 µM	103
15	CWRDYLIX	Cys-AcrK bridge<br>X = AcrK	TEV protease	8.2 µM	105
16	CQWFSHRX	Cys-AcrK bridge<br>X = AcrK	TEV protease	6.9 µM	105
17	CQSLWMNX	Cys-AcrK bridge<br>X = AcrK	HDAC8	7.1 µM	105
18	CFIHPQGDX	Cys-O2beY bridge<br>X = O2beY	Streptavidin	150 nM	106
19	CWWHPQGDX	Cys-O2beY bridge<br>X = O2beY	Streptavidin	20 nM	106
20	CWMHPQGDX	Cys-O2beY bridge<br>X = O2beY	Streptavidin	550 nM	106
21	CWTHPQFDX	Cys-O2beY bridge<br>X = O2beY	Streptavidin	425 nM	106
22	XDSETGEC	Cys-O2beY bridge<br>X = O2beY	Keap1Kelch	110 nM	106
23	XDVETGEC	Cys-O2beY bridge<br>X = O2beY	Keap1Kelch	425 nM	106
24	GSSPXEAMDMCTDTG	Cys-O2beY bridge<br>X = O2beY	Sonic Hedgehog	550 nM	106
25	HVGPXEAMDMCTDTG	Cys-O2beY bridge<br>X = O2beY	Sonic Hedgehog	1.1 µM	106
26	LSGPXEAMDMCTDTG	Cys-O2beY bridge<br>X = O2beY	Sonic Hedgehog	3.7 µM	106
27	GSMVXEAMDMCTDTG	Cys-O2beY bridge<br>X = O2beY	Sonic Hedgehog	3.7 µM	106
28	XDIETGEC	Cys-O2beY bridge<br>X = O2beY	Keap1Kelch	43 nM	106

29	XDAETGEC	Cys-O2beY bridge X = O2beY	Keap1Kelch	40 nM	106
30	XSRDWRFC	Cys-O2beY bridge X = O2beY	Sonic Hedgehog	4.9 µM	106
31	XTKQSSNC	Cys-O2beY bridge X = O2beY	Sonic Hedgehog	11.5 µM	106
32	XRSKTFEC	Cys-O2beY bridge X = O2beY	Sonic Hedgehog	6.1 µM	106
33	XYSDTLQC	Cys-O2beY bridge X = O2beY	Sonic Hedgehog	5.6 µM	106
34	SWIVP	sulfonamide modified N-terminus	Carbonic anhydrase	6.7 nM	130
35	SWNTK	sulfonamide modified N-terminus	Carbonic anhydrase	~ 7 nM	130
36	SWYKL	sulfonamide modified N-terminus	Carbonic anhydrase	~ 10 nM	130
37	SWQQQ	sulfonamide modified N-terminus	Carbonic anhydrase	~ 15 nM	130
38	SWPAR	sulfonamide modified N-terminus	Carbonic anhydrase	~ 15 nM	130
39	SFVVR	sulfonamide modified N-terminus	Carbonic anhydrase	~ 15 nM	130
40	SYQYR	sulfonamide modified N-terminus	Carbonic anhydrase	~ 30 nM	130
41	SYQYS	sulfonamide modified N-terminus	Carbonic anhydrase	~ 40 nM	130
42	STRPA	sulfonamide modified N-terminus	Carbonic anhydrase	34 nM	130
43	SWTSG	sulfonamide modified N-terminus	Carbonic anhydrase	~ 50 nM	130
44	SWTWL	sulfonamide modified N-terminus	Carbonic anhydrase	~ 55 nM	130
45	SWTYW	sulfonamide modified N-terminus	Carbonic anhydrase	~ 140 nM	130
46	SAHPL	Biotinylated N-terminus	Streptavidin	114 fM	130
47	SHFTN	Biotinylated N-terminus	Streptavidin	57 fM	130
48	SGYTQ	Biotinylated N-terminus	Streptavidin	49 fM	130
49	SANSSFAP	Ara_6-modified N-terminus	CS-35 Ab	1.4 µM	131
50	STTYVVNP	Ara_6-modified N-terminus	CS-35 Ab	1.9 µM	131
51	SSAHNTMS	Ara_6-modified N-terminus	CS-35 Ab	3.2 µM	131
52	SDAHATLR	Ara_6-modified N-terminus	CS-35 Ab	7 µM	131
53	SITHAPS	Mannose-modified N-terminus	DC-SIGN	450 µM	132

54	SWKPE	Galactose-modified N-terminus	Galectin-3	~ 8 mM[a]	133
55	SLSMA	Galactose-modified N-terminus	Galectin-3	~ 9 mM[a]	133
56	SWHVP	Galactose-modified N-terminus	Galectin-3	~ 10 mM[a]	133
57	ACVSPRSHECGGG	APBA-modified cysteines	*S. aureus*	1.5 µM	139
58	ACTLPNGPRCGGG	APBA-modified cysteines	*A. baumannii*	0.3 µM	139
59	ACDPNRMDRCGGG	APBA-modified cysteines	*mcr-1* positive *E. coli*	n.d.	141
60	ACMTNTPVPCGGG	APBA-modified cysteines	*mcr-1* positive *E. coli*	n.d.	141
61	ACRAHEQSLCGGG	APBA-modified cysteines	*mcr-1* positive *E. coli*	n.d.	141
62	ACSERQHLQCGGG	APBA-modified cysteines	*Klebsiella pneumonia* 497	n.d.	140
63	ACRSHDSAMCGGG	APBA-modified cysteines	*Klebsiella pneumonia* 497	n.d.	140
64	ACLATKGSICGGG	APBA-modified cysteines	*Klebsiella pneumonia* 497	n.d.	140
65	ACKPLHSRSCGGG	APBA-modified cysteines	*A. baumannii* EGA408	n.d.	140
66	ACTNANHYFCGGG	APBA-modified cysteines	*A. baumannii* EGA408	n.d.	140
67	ACYSSPSHFCGGG	APBA-modified cysteines	*A. baumannii* EGA408	n.d.	140
68	CRPLGENLETC	APBA-modified N-terminal cysteine α-cyanoacrylamide-modified internal cysteine	TEV Protease	1.8 µM	146
69	CTPFGENLMYC	APBA-modified N-terminal cysteine α-cyanoacrylamide-modified internal cysteine	TEV Protease	2.0 µM	146
70	CFEIGDNLMSC	APBA-modified N-terminal cysteine α-cyanoacrylamide-modified internal cysteine	TEV Protease	4.9 µM	146
71	CKEYGHNLLKC	APBA-modified N-terminal cysteine α-cyanoacrylamide-modified internal cysteine	TEV Protease	3.5 µM	146

72	CTSLNENLLYC	APBA-modified N-terminal cysteine α-cyanoacrylamide-modified internal cysteine	TEV Protease	3.0 μM	146
73	CTTVSDELEYC	APBA-modified N-terminal cysteine α-cyanoacrylamide-modified internal cysteine	TEV Protease	6.9 μM	146
74	GPYPECILDCHVQSVWT	Cysteines cyclized with mDBMB or CDCB	β-catenin	36 nM (mDBMB) 26 nM (CDCB)	155
75	GAYPECILDCHVARVQW	Cysteines cyclized with mDBMB or CDCB	β-catenin	13 nM (mDBMB) 5.4 nM (CDCB)	155
76	GGYPECILDCHLQRVIL	Cysteines cyclized with mDBMB or CDCB	β-catenin	8.4 nM (mDBMB) 7.1 nM (CDCB)	155
77	GGWPECILDCHVQRVWQ	Cysteines cyclized with mDBMB or CDCB	β-catenin	16 nM (mDBMB) 5.2 nM (CDCB)	155
78	GGWPECILDCHVARVWS	Cysteines cyclized with mDBMB or CDCB	β-catenin	5.6 nM (mDBMB) 7.9 nM (CDCB)	155
79	HCCKWYDFCIECI	Cysteines double-bridged by DBAc	IL-17	75 nM	156
80	VCCDYWYGFCDLCP	Cysteines double-bridged by DBAc	IL-17	36 nM	156
81	SCCQGCRVLCY	Cysteines double-bridged by pDBMB	Kallikrein	0.5 nM	156
82	TCCRGCRVLCY	Cysteines double-bridged by pDBMB	Kallikrein	0.7 nM	156
83	GCTRQVCQQTPACPSACN	Cysteines double-bridged by mDBMP	Kallikrein-related peptidase 5	7 nM	158
84	GCTRQWPCDSVPGSCTCV	Cysteines double-bridged by mDBMP	Kallikrein-related peptidase 5	2.0 nM	158
85	ACTREYNPQCANPQCLCH	Cysteines double-bridged by mDBMP	Kallikrein-related peptidase 5	6 nM	158
86	GCTRQWPCDSVPGSCTCV	Cysteines double-bridged by DBAc	Kallikrein-related peptidase 5	2.7 nM	158

87	ACTREYNPQANPQCLCH	Cysteines double-bridged by DBAc	Kallikrein-related peptidase 5	28 nM	158
88	MCSRQWDPSLCCPQRTCP	Cysteines double-bridged by DBAc	Kallikrein-related peptidase 5	25 nM	158
89	RCDSSQCMQGQCLYRSCP	Cysteines double-bridged by mDBMP	Kallikrein-related peptidase 7	94 nM	158
90	HCRKGLDRCPQCLYRTCS	Cysteines double-bridged by mDBMP	Kallikrein-related peptidase 7	66 nM	158
91	QCLYIACPSAAKCDNGCQ	Cysteines double-bridged by mDBMP	Kallikrein-related peptidase 7	32 nM	158
92	QCLYMARCSPVCPQQYCQ	Cysteines double-bridged by DBAc	Kallikrein-related peptidase 7	9 nM	158
93	QCIITYQFCTHDRYGCCS	Cysteines double-bridged by DBAc	Kallikrein-related peptidase 7	96 nM	158
94	SICRFFC	Cysteines bridged by DFS	HSA	3.5 µM	160
95	CEFLDWEMDGC	Cysteines bridged by DFB	Bcl-xl	1.2 µM	161
96	ACGFERERTCG	Cysteines bridged by BSBCA	Streptavidin	452 µM (trans) >2000 µM (cis)	138
97	ACLSQRDGNCG	Cysteines bridged by BSBCA	Streptavidin	368 µM (trans) 814 µM (cis)	138
98	ACSVKLHTHCG	Cysteines bridged by BSBCA	Streptavidin	506 µM (trans) 1223 µM (cis)	138
99	AGCWQAWTCVG	Cysteines bridged by BSBBA	Streptavidin	3.4 µM (trans) 1.8 µM (cis)	162
100	AGCWGQWACQG	Cysteines bridged by BSBBA	Streptavidin	6.7 µM (trans) 2.2 µM (cis)	162
101	ASCGPSFFCNG	Cysteines bridged by BSBBA	Streptavidin	3.1 µM (trans) 2.1 µM (cis)	162
102	ASCFPRWVCGG	Cysteines bridged by BSBBA	Streptavidin	3.6 µM (trans)	162

				2.0 µM (cis)	
103	SFCDTYC	Cysteines bridged by DPD and modified with sulfonamide	Bovine carbonic anhydrase	41 nM	165
104	SICFDYC	Cysteines bridged by DPD and modified with sulfonamide	Bovine carbonic anhydrase	40 nM	165
105	ACFMQEPLYICG	Cysteines bridged by DCA-VS	TEV Protease	730 nM	166
106	VCARWRWMPCG	Cysteines bridged by DCA-DPP	FphF	650 nM	166
107	CPFPPSWC	Cysteines bridged by DCA	Sortase A	22 µM	167
108	CPFPASWC	Cysteines bridged by an APBA-modified dichloro-oxime	Sortase A	2.9 µM	167
109	CYYNRSGEYVC	Cysteines bridged by an APBA-modified dichloro-oxime	SARS-CoV-2 Spike RBD	1.2 µM	167
110	CPVRYGWDMRC	Cysteines bridged by ClAc-3	Bcl-2	0.716 µM	145
111	CPARYGWDYEC	Cysteines bridged by ClAc-3	Bcl-2	0.365 µM	145
112	CPIRYPWDRVC	Cysteines bridged by ClAc-3	Bcl-2	0.626 µM	145
113	CTFELYWDGLC	Cysteines bridged by ClAc-3	MDM2	104 nM	145
114	CSFRSYWDGLC	Cysteines bridged by ClAc-3	MDM2	143 nM	145
115	CDPETGEQAEC	Cysteines bridged by ClAc-3	Keap1	45 nM	145
116	CIDPETGEEQC	Cysteines bridged by ClAc-3	Keap1	219 nM	145
117	CVPESARSDDDGEC	Cysteines bridged by CAmCBT	SARS-CoV-2 Spike RBD	5 µM	143
118	CQLDSETGEKC	Cysteines bridged by M-a-23	Keap1 Kelch	60 nM	144
119	CSYRDNETGEC	Cysteines bridged by M-a-23	Keap1 Kelch	140 nM	144
120	CASILPPYTSC	Cysteines bridged by M-a-23	Sortase A	16 µM	144
121	CSHLLPPYKSC	Cysteines bridged by M-a-23	Sortase A	18 µM	144
122	AWWTNDFCACA	N-terminus to cysteine bridge by alkylation/reduction	Streptavidin	71 nM	168
123	ACSDRFRNCPADEALCG	Bicylic peptide bridged by TBMB	Kallikrein	20 nM	169
124	NCKFSGCSVSVCH	Bicylic peptide bridged by TBMB	Serine protease uPA	5.1 µM	170

125	NCKFSGCPWELC	Bicylic peptide bridged by TBMB	Serine protease uPA	0.78 µM	170
126	TCVNIMCCRCP	Cysteines double-bridged with DBAc	FXIa	19 nM	171
127	GCTCWECWWLCS	Cysteines double bridged with DBAc	IL-23R	310 nM	171
128	ACAQKLDGCSYISWSCG	Bicylic peptide bridged by TBMB	β-catenin	4.57 µM	175
129	ACSGWWPKCQGYIPGCG	Bicylic peptide bridged by TBMB	β-catenin	3.8 µM	175
130	ACAPGVYRCNQNFIWC	Bicyclic peptide bridged by TATA	β-catenin	3.9 µM	175
131	ACPAVGEDCVFNWAVCG	Bicyclic peptide bridged by TBAB	β-catenin	6.3 µM	175
132	GCGGRPCPPAYCG	Bicyclic peptide bridged by TBMB	FXIIa	1.2 µM	176
133	ACHSRCPQLPPCG	Bicyclic peptide bridged by TBMB	*S. aureus* Sortase A	1.1 µM	177
134	ACPLLPPCSLDCG	Bicyclic peptide bridged by TBMB	*S. aureus* Sortase A	1.5 µM	177
135	ACTSRCPQLPPCG	Bicyclic peptide bridged by TBMB	*S. aureus* Sortase A	1.8 µM	177
136	RCAGPVCPWTRCG	Bicylic peptide bridged by TATA	Serine protease uPA	0.8 µM	178
137	GCFPSYCPQVACQ	Bicyclic peptide bridged by TBAB	Serine protease uPA	0.78 µM	178
138	RCAGPACPWQRCS	Bicyclic peptide bridged by TATA	Serine protease uPA	93 nM	178
139	RCAGPACPWQKCS	Bicyclic peptide bridged by TATA	Serine protease uPA	85 nM	178
140	ACSLQDPNCDWWGPYCG	Bicyclic peptide bridged by TBMB	Her2	460 nM	179
141	ACYLQDPNCDWWGHYCG	Bicyclic peptide bridged by TBMB	Her2	820 nM	179
142	ACYLQDPNCDWWGPYCG	Bicyclic peptide bridged by TBMB	Her2	300 nM	179
143	ARDCPLVNPLCLHPGWTCA	Bicyclic peptide bridged by TBMB	EphA2	5.8 nM	182
144	GACPPCVWQVFCGGSGA	Bicyclic peptde bridged by TBMB-Me	TNFα	7.6 nM	185
145	SYCKRAHKNC	Bicyclic peptide bridged by TSL-6	NODAL	<10 µM	172

[a]Peptide affinity not significantly higher than galactose control, but phages showed selective affinity for Galectin-3
